# Functional switching from circularly polarized luminescence to whispering gallery modes in a chiral Schiff base

**DOI:** 10.1016/j.isci.2026.117379

**Published:** 2026-08-29

**Authors:** Jinhui Li, Yue Luo, Cong Chen, Liang Tong, Jiabin Qiu, Soh Kushida, Yohei Yamamoto

**Affiliations:** 1 College of Chemistry and Chemical Engineering, Jiangxi Provincial Key Laboratory of Functional Crystalline Materials Chemistry, Jiangxi University of Science and Technology, Ganzhou 341000, China; 2 Department of Materials Science, Institute of Pure and Applied Sciences, University of Tsukuba, 1-1-1 Tennodai, Tsukuba, Ibaraki 305-8573, Japan; 3 Tsukuba Research Center for Energy Materials Science (TREMS), Hydrogen Boride Research Center, Tsukuba Institute for Advanced Research, University of Tsukuba, 1-1-1 Tennodai, Tsukuba, Ibaraki 305-8573, Japan

**Keywords:** whispering gallery mode, circularly polarized luminescence, functional switching, chiral materials

## Abstract

This work demonstrates a design concept for dual-mode optical materials, embodied by an axially chiral fluorescent Schiff base, (*R*/*S*)-BN-2HGCA. The compound exhibits two competing optical functions that are toggled by its physical aggregation state. In a dilute solution, the molecule’s chirality is expressed, resulting in clear circularly polarized luminescence (CPL). In contrast, upon self-assembly into morphologically pristine microspheres, the chiroptical property is suppressed. This structural transition represents a functional transition where intense optical confinement within the microcavity is accompanied by suppression of the observable CPL, while enabling the emergence of whispering gallery mode (WGM) resonance. These spheres function as active optical resonators supporting distinct WGM resonance, with an average Q-factor of approximately 190, demonstrating effective light confinement within the self-assembled organic microcavities. Controlling dissolution and self-assembly to switch between chiroptical and photonic modalities provides a promising design concept that may be developed toward smart materials.

## Highlights

• A chiral Schiff base switches between chiroptical and photonic states
• Dissolved enantiomers emit mirror-image circularly polarized light
• Self-assembly into amorphous microspheres suppresses chiral emission
• Microspheres support whispering-gallery resonances with a quality factor of ∼190

## Graphical abstract

**Figure:**
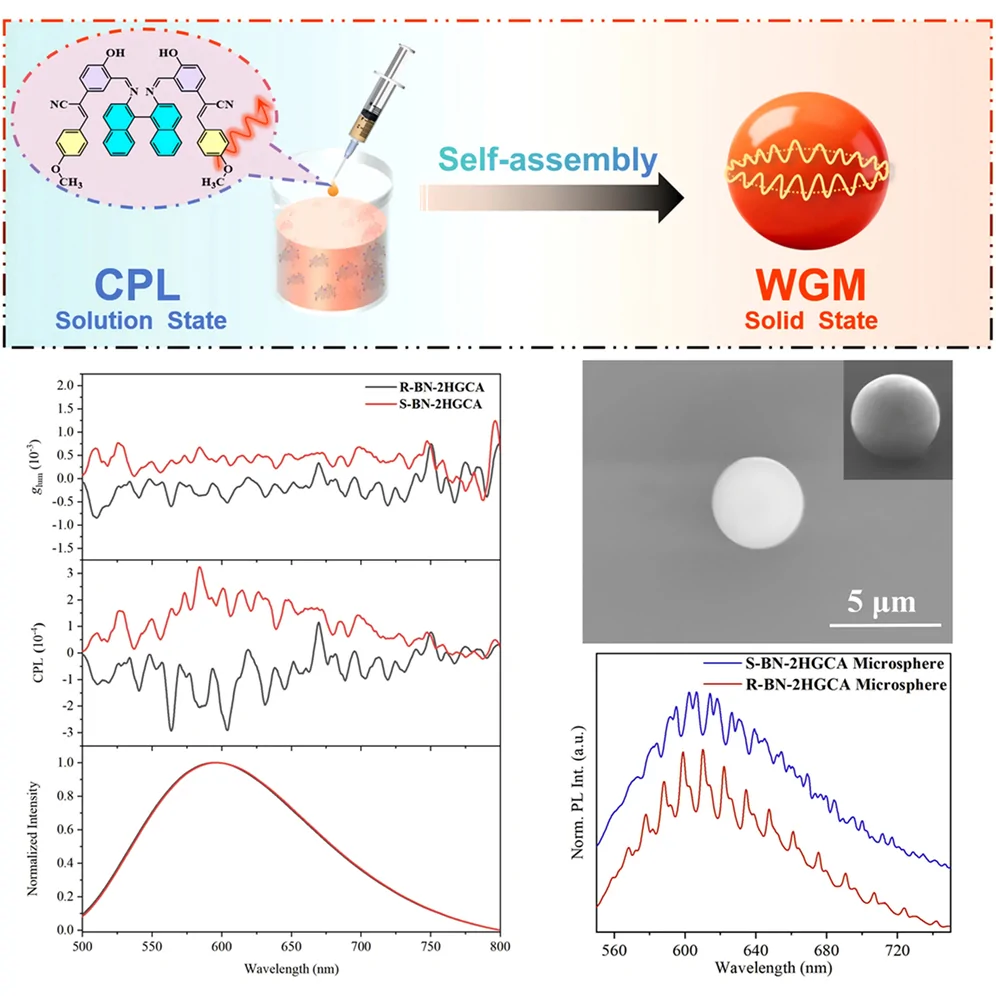

## Teaser

Physics; Condensed matter physics; Applied sciences

## Introduction

The design of smart materials whose optical properties can be switched by external stimuli is a central theme in materials science. Advanced applications now demand materials that not just have “ON-OFF” modes but are multi-modal, capable of switching between entirely different, complex functional states.^1^^,^^2^ Among these, circularly polarized luminescence (CPL) represents a sophisticated chiroptical property that describes the differential emission of left- and right-handed circularly polarized light. This property encodes molecular-level chiral information for use in 3D displays and information encryption.^3^^,^^4^

Concurrently, whispering gallery mode (WGM) resonators represent a powerful photonic mode arising from the total internal reflection of light within a symmetric microcavity. This structural perfection at the micrometer scale enables intense light confinement for microlasers and sensing.^5^^,^^6^ Integrating these two advanced optical modalities is a formidable challenge.^7^^,^^8^ This difficulty stems from their conflicting structural prerequisites. CPL typically benefits from long-range chiral order, whereas high-Q WGMs rely on morphological perfection and an amorphous, isotropic interior to minimize scattering.^9^^,^^10^ Significant efforts have been made to harness these distinct properties. For instance, some researchers have engineered complex chiral photonic crystal slabs to create microcavities that force circularly polarized emission.^11^^,^^12^ Separately, the inherent sensitivity of WGMs to morphology has been ingeniously exploited. Yamamoto and co-workers, for example, utilized self-assembled amorphous diarylethene microresonators to create unreplicable “optical fingerprints.” In their system, each amorphous pixel possessed a unique WGM spectrum, creating a physical unclonable function (PUF) for high-security authentication.^13^ While these examples show the advanced applications of individual optical activity, developing one-component material that can be deliberately toggled between CPL and WGM states remains a significant goal through simple self-assembly.^14^^,^^15^

A pathway can be developed where two functional states are self-assembled from a single material and can be switched. Here, we demonstrate functional switching between CPL and WGM by using a novel, axially chiral fluorescent Schiff base, (*R*/*S*)-BN-2HGCA. In solution, the chirality of (*R*/*S*)-BN-2HGCA is reflected to clear CPL signal, with |*g*_lum_| value of 4.5 × 10^−4^. Upon self-assembly into morphologically pristine microspheres, the molecular-level chiroptical property is suppressed, but this structural change simultaneously unlocks the emergence of WGM resonance. This functional transition illustrates a design concept for dual-mode materials, where a simple external trigger toggles the system between its chiroptical and photonic states. This offers a conceptual framework that could inspire future explorations toward applications such as multi-level anti-counterfeiting and high-contrast sensing.^16^^,^^17^

## Results

### Synthesis and chiroptical properties of (*R*/*S*)-BN-2HGCA

**Figure 1:**
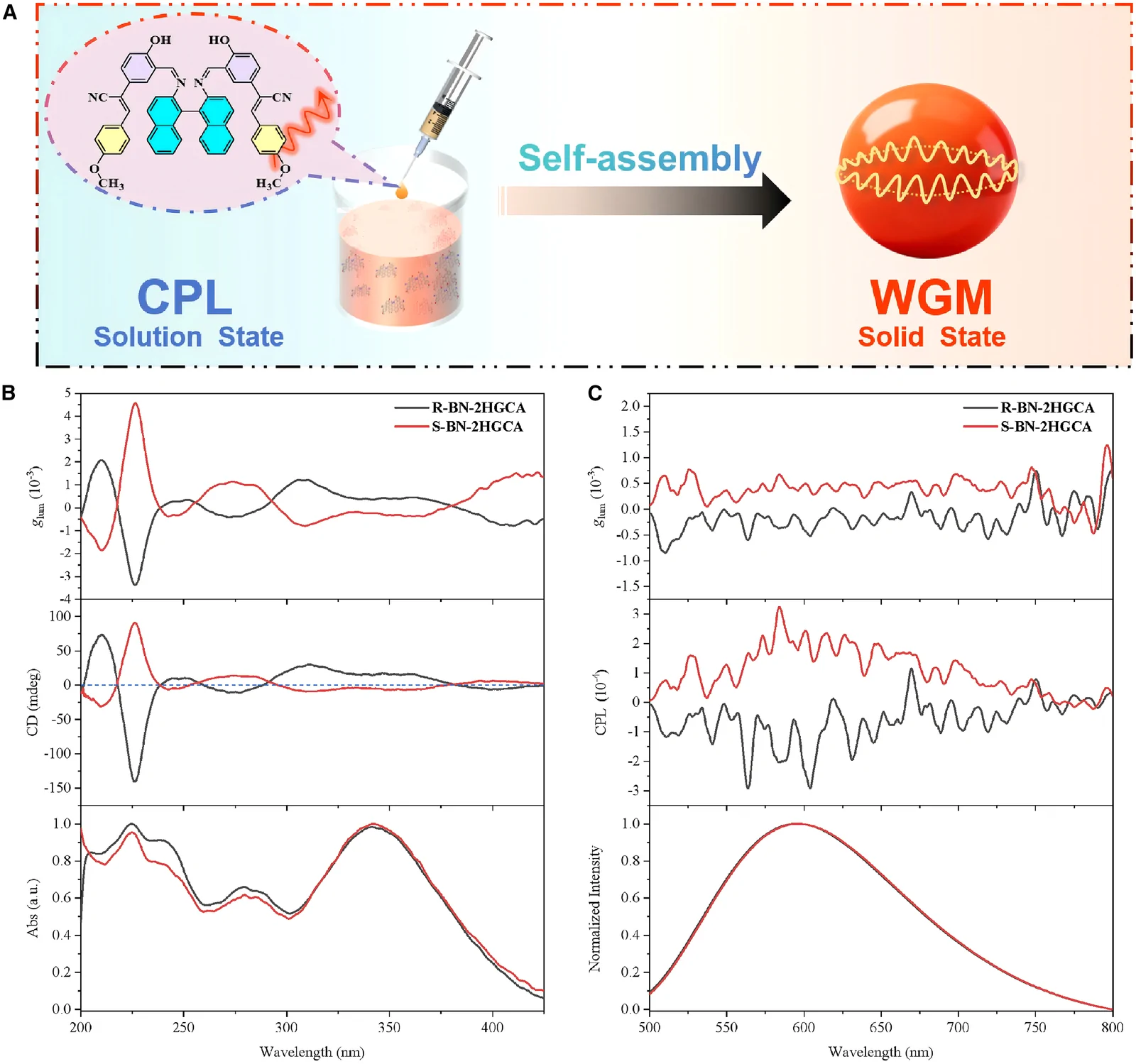
Molecular design and solution-state properties (A) Conceptual illustration of the (*R*/*S*)-BN-2HGCA molecule. (B and C) CD spectra (B) and CPL spectra (C) of (*R*/*S*)-BN-2HGCA in methanol solution.

The design strategy centered on creating a single, multifunctional molecule that integrates three key components, as illustrated in the conceptual diagram in Figure 1A. The (*R*/*S*)-[1,1′-binaphthalene]-2,2′-diamine (BINAM) scaffold was selected as the foundational chiral unit, as its C_2_-symmetric backbone is well known for stable axial chirality and its ability to effectively transfer stereochemical information.^18^ The luminophore component was constructed from a cyanostilbene derivative, selected to provide an extended π-conjugation and serve as the core emissive unit.^19^^,^^20^ Finally, a Schiff base linkage was used to covalently connect the chiral scaffold to the luminophore arms. This design aims to (1) induce and lock a twisted, chiral conformation in the achiral luminophore arms via the rigid BINAM core, and (2) provide hydroxyl and cyano groups that can act as hydrogen-bonding sites to direct supramolecular self-assembly.

The synthesis of the target molecule (*R*/*S*)-BN-2HGCA is outlined in Scheme S1. The structures of all intermediates and final products were rigorously characterized by ^1^H NMR and Fourier transform infrared spectroscopy (FTIR) (see Figures S1 and S2). The ground-state chiroptical properties were evaluated first (Figure 1B). The circular dichroism (CD) spectra of the (*R*)- and (*S*)-enantiomers in methanol solution exhibit a clear mirror-image relationship, confirming their enantiomeric purity. The influence of the BINAM core is evident, with a prominent bisignate signal observed between 220 and 250 nm, corresponding to the electronic transitions of the naphthalene units.^21^ Furthermore, distinct Cotton effects are visible at the longer wavelengths. This observation provides crucial validation for the molecular design, demonstrating that the ground-state chirality has successfully been transferred from the BINAM framework to the entire molecule, thereby enabling the formation of a twisted chiral conformation.

The corresponding UV-vis absorption spectra are presented in Figure 1B. As expected for enantiomers, their spectra are identical, displaying complex absorption bands across the 250–450 nm range. A high-energy absorption maximum is observed at approximately 230 nm, while a broad, lower-energy absorption band is centered at approximately 340 nm. These absorption bands directly overlap with the observed Cotton effects in the CD spectra, confirming that the chiroptical signals originate from the molecule’s electronic transitions, including those of the chirally perturbed luminophore arms.^22^^,^^23^

The electronic transitions in this system are also sensitive to the solvent environment. As shown in Figure S4, when measured in pure N,N-dimethylformamide, the low-energy absorption band exhibits a significant bathochromic shift, with a new maximum observed at 367 nm. This solvatochromism, a shift of 27 nm relative to methanol, suggests that the transition possesses intramolecular charge transfer (ICT) character, with an excited state that is more stabilized by polar solvents than the ground state.^23^ The fundamental (achiral) excited-state properties in chloroform were characterized by photoluminescence (PL) spectroscopy (Figure S5). The excitation spectrum is broad, consistent with the multiple absorption bands observed in Figure 1B. Upon excitation, both the (*R*)- and (*S*)-enantiomers exhibit identical, single-peak fluorescence emission profiles centered at approximately 600 nm. The perfect overlap of the R and S emission spectra is the physically expected result for enantiomers in an achiral solvent. Following the ground-state analysis, the expression of this chirality in the excited state was investigated. The excited-state properties were evaluated in methanol solution. The CPL spectra are presented in Figure 1C. The (*R*)- and (*S*)-enantiomers display distinct mirror-image CPL signals in the fluorescence region, albeit with a low signal-to-noise ratio.

This excited-state chiroptical activity is quantified by the luminescence dissymmetry factor (*g*_lum_), shown in Figure 1C. The *g*_lum_ spectra confirm the mirror-image relationship. Taking the average of 550–700 nm, the (*S*)-enantiomer shows a positive *g*_lum_ of +4.5 × 10^−4^, while the (*R*)-enantiomer shows a negative *g*_lum_ of −2.1 × 10^−4^. This quantitative chiroptical response confirms that (*R*/*S*)-BN-2HGCA is a CPL-active material in its dissolved state. This establishes a “CPL-ON” baseline and verifies that the molecular chirality is successfully translated into chiral light emission. The reported values are averaged over the 550–700 nm emission band. The (*R*)- and (*S*)-enantiomers exhibit mirror-image CPL of opposite sign, as expected for enantiomers. To examine the difference in absolute magnitude, the CPL was measured in five different solvents—toluene, tetrahydrofuran (THF), *N*,*N*-dimethylformamide (DMF), acetone, and 1,4-dioxane (Figure S10); in every solvent, the two enantiomers give *g*_lum_ of opposite sign, with a clear mirror-image relationship, while the mean of the two enantiomers is consistently offset from zero by approximately +1 × 10^−4^, independent of the solvent, concentration, and sample. We interpret this constant, solvent-independent offset as a small baseline offset inherent to the weak-signal measurement, rather than an intrinsic chiroptical asymmetry; once it is taken into account, the (*R*) and (*S*) responses are comparable in magnitude. We, therefore, base our conclusions on the reproducible sign and mirror-image character of the CPL, rather than on the precise absolute magnitudes. A *g*_lum_ on the order of 10^−4^ is typical for molecularly dispersed axially chiral small-molecule emitters (Table S1) and is sufficient to define the CPL-ON baseline.

### Morphology and chiroptical suppression in the amorphous state

**Figure 2:**
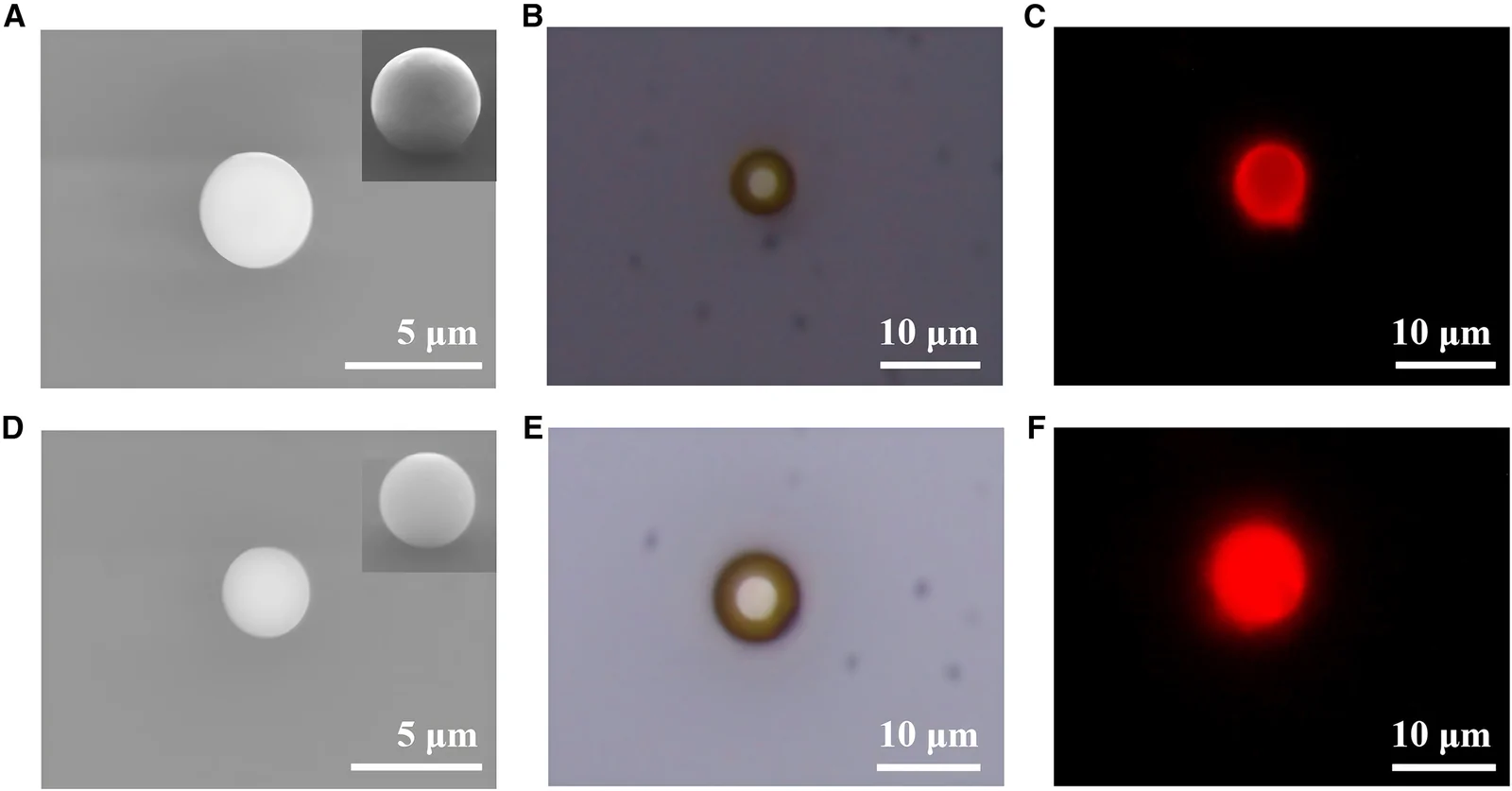
Morphological and optical characterization of self-assembled microspheres (A–C) SEM image, (B) OM image, and (C) FM image of (*R*)-enantiomer microspheres; inset shows a high-magnification tilted view. (D–F) SEM image, (E) OM image, and (F) FM image of (*S*)-enantiomer microspheres; inset shows a high-magnification tilted view.

The (*R*/*S*)-BN-2HGCA molecules were self-assembled into microstructures, using a miniemulsion method (see Scheme S2).^24^ This process yielded a high-density population of microspheres, as evidenced by the scanning electron microscopy (SEM) images in Figure 2. The particles produced from both the (*R*)- and (*S*)-enantiomers (Figures 2A and 2D) are morphologically indistinguishable. They appear as highly uniform, well-defined spheres. The high-magnification tilted images (insets of Figures 2A and 2D) reveal that the spheres possess a notably smooth surface without visible grain boundaries, cracks, and crystalline facets. Bright-field optical microscopy (OM), shown in Figures 2B and 2E, confirms the presence of these well-defined spherical particles. As shown in Figures 2C and 2F, in fluorescence microscopy (FM) testing, the microspheres exhibit bright, uniform red-orange fluorescence across their entire volume when excited at 405 nm. This observation directly correlates the fluorescent molecule with the microspherical morphology, confirming that the spheres are self-assembled aggregates of the (*R*/*S*)-BN-2HGCA molecule.

**Figure 3:**
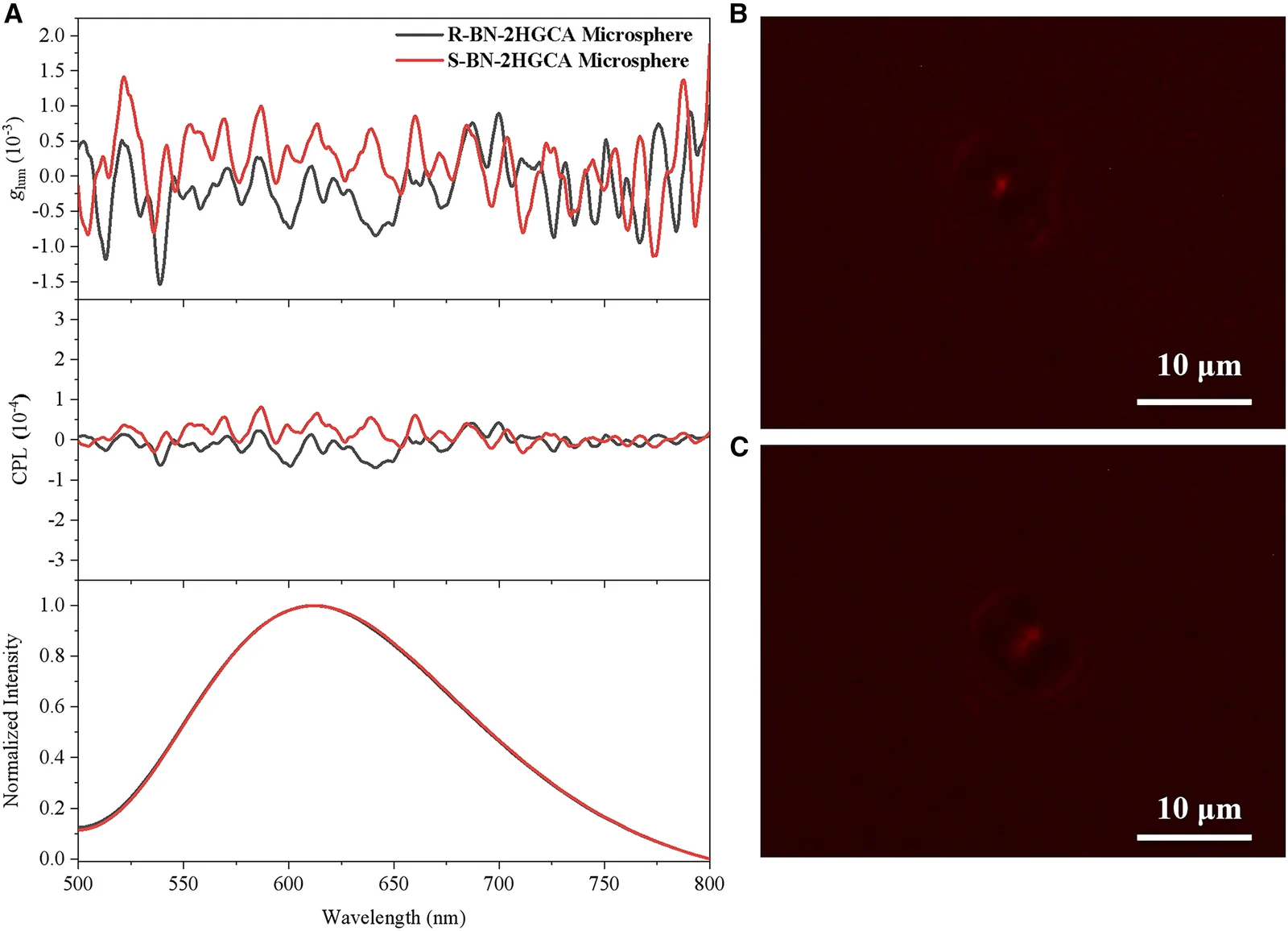
Chiroptical properties of microspheres in the aggregated state (A) CPL spectra of (*R*)- and (*S*)-enantiomer microspheres dispersed in water. (B and C) POM image of (B) (*R*)-enantiomer microspheres and (C) (*S*)-enantiomer microspheres.

Figure 3A displays the CPL spectrum of the (*R*)- and (*S*)-enantiomer microspheres dispersed in water. In stark contrast to the CPL signals observed in the solution state (Figure 1C), the microsphere dispersion exhibits no discernible chiroptical activity, with the CPL spectrum consisting only of baseline noise. This is quantitatively confirmed by the *g*_lum_ spectrum, where the signal oscillates randomly around zero. The observed peak values, at approximately ±0.002, are characteristic of the instrument’s noise floor and do not represent a true chiroptical signal, demonstrating a complete suppression of the CPL function upon aggregation and an effective switch from a “CPL-ON” to a “CPL-OFF” state. To understand the origin of this functional suppression, the internal structure of the microspheres was probed, proceeding with the hypothesis that the molecules adopted a disordered, amorphous packing. Polarized optical microscopy (POM) analysis (Figures 3B and 3C) provides strong support for this hypothesis. After a uniform adjustment of image contrast and color temperature applied equally to the entire field of view, the interior of the microspheres remains dark, with only weak residual brightness at the edges attributable to boundary refraction, exhibiting no characteristic Maltese cross or other birefringence patterns associated with crystalline or liquid crystalline spherulites.^25^ The very faint light visible at the edges is attributed to light scattering from the curved surface rather than intrinsic birefringence.^26^ This pronounced lack of optical anisotropy is a clear indicator of an amorphous, glassy internal structure. The macroscopic suppression of the observable CPL in the aggregated state likely arises from a combination of factors related to the aggregated structure. In the solution state, individual chiral molecules freely adopt their CPL-active conformations to emit circularly polarized light. However, upon assembling into morphologically pristine microspheres, the emitted light undergoes multiple total internal reflections at the highly refractive interface. In addition, upon assembling into morphologically pristine microspheres, the emitted light undergoes multiple total internal reflections at the highly refractive interface; this optical confinement, which serves as the prerequisite for WGM resonance, may further randomize the polarization state of the confined light. We regard this cavity-related depolarization as a hypothesis consistent with the present observations, rather than a directly proven, exclusive mechanism.^27^ Consequently, the observable CPL signal is suppressed in the aggregated state, accomplishing a physical functional switch. Disentangling the relative contributions of the disordered molecular packing and the cavity-induced polarization randomization—for example, by embedding the microspheres in a refractive-index-matching medium to suppress the internal reflections—is an informative direction for future work.

### Characteristic analysis of WGM

**Figure 4:**
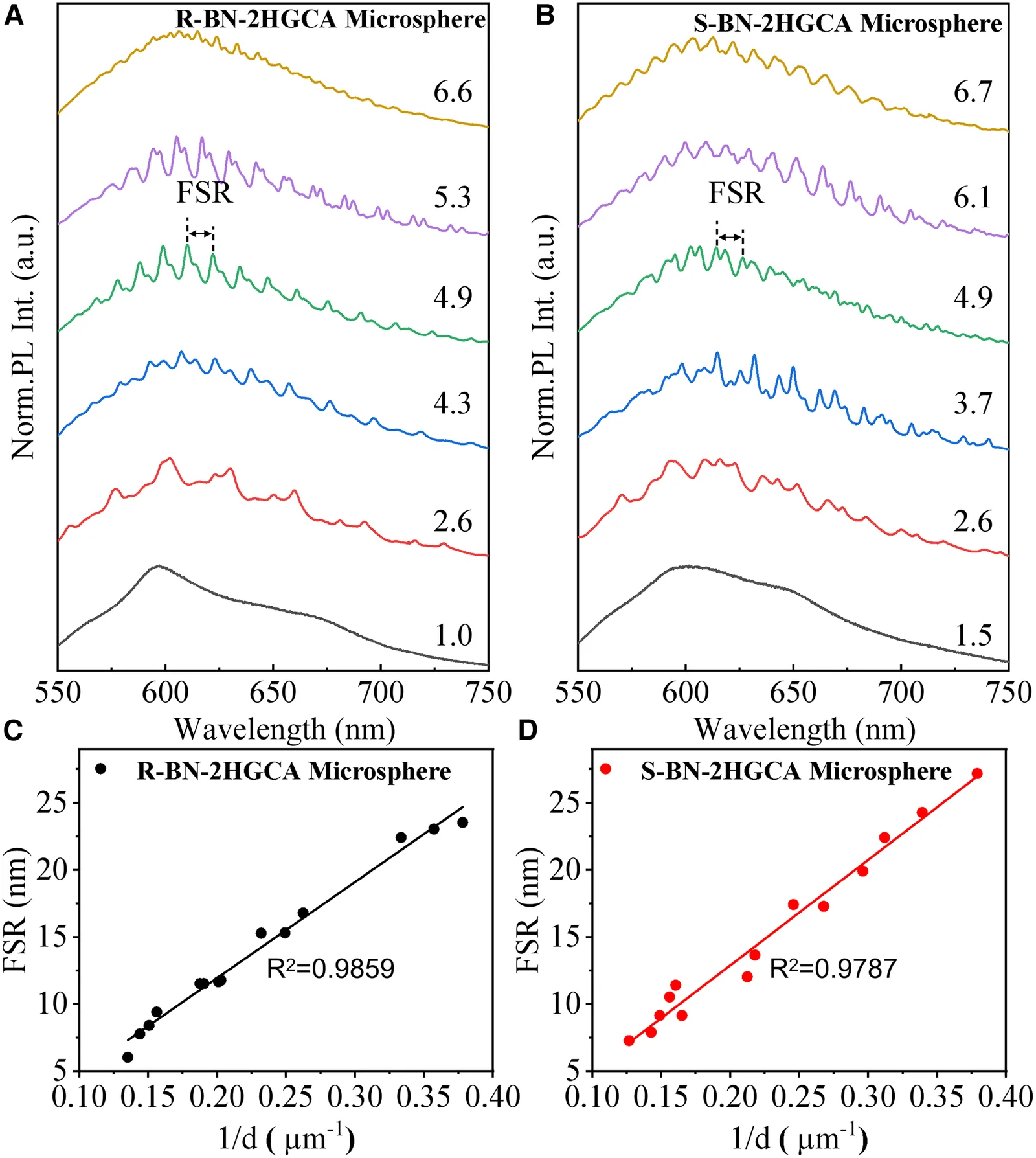
WGM and size dependence (A and B) μ-PL spectra of individual microspheres with different diameters, showing clear WGM resonance. (C and D) Linear plot of the FSR versus the inverse of the microsphere diameter (1/d).

The structural properties of the microspheres, specifically their smooth surfaces and amorphous interiors as established in the previous section, are ideal prerequisites for optical microcavities.^28^^,^^29^ This hypothesis was investigated by performing micro-photoluminescence (μ-PL) spectroscopy on single, isolated spheres. Here, μ-PL is a spontaneous-emission, low-excitation-density measurement that probes the passive optical modes of a single resonator and is, therefore, distinct from optically pumped lasing, which requires reaching a population-inversion threshold under high pump fluence; our analysis is confined to passive WGM resonances in the fluorescence spectra and does not assert laser action. A dramatic functional transformation is observed. In contrast to the broad, featureless emission in solution, the μ-PL spectra from individual microspheres are modulated into a periodic series of sharp, discrete resonant peaks (Figures 4A and 4B). These peaks are the unambiguous signature of WGMs.^30^^,^^31^ This phenomenon arises from the confinement of fluorescent light by total internal reflection at the smooth, high-refractive-index boundary of the spherical particle. As this light circulates, only wavelengths that satisfy the constructive interference condition are resonantly enhanced, emerging as the sharp peaks. The WGM phenomenon is further confirmed by the systematic size dependence shown in Figures 4A and 4B. A clear trend is visible, where the spacing between the peaks, known as the free spectral range (FSR), increases as the microsphere diameter (*d*) decreases. Theoretically, this trend follows Equation 1:

(Equation 1) $$\begin{array}{c} FSR=\lambda^{2}/\left(n\pi d\right) \end{array}$$

where *n* is the refractive index of the medium.^32^

This inverse relationship between FSR and particle diameter is a fundamental physical characteristic of WGM resonance.^33^ To provide definitive and quantitative proof, the FSR was extracted for multiple spheres and plotted against the inverse diameter, as shown in Figures 4C and 4D. The data points form a clear linear relationship, which is the proof of the WGM phenomenon and confirms that the emission is governed by photonic resonance.

**Figure 5:**
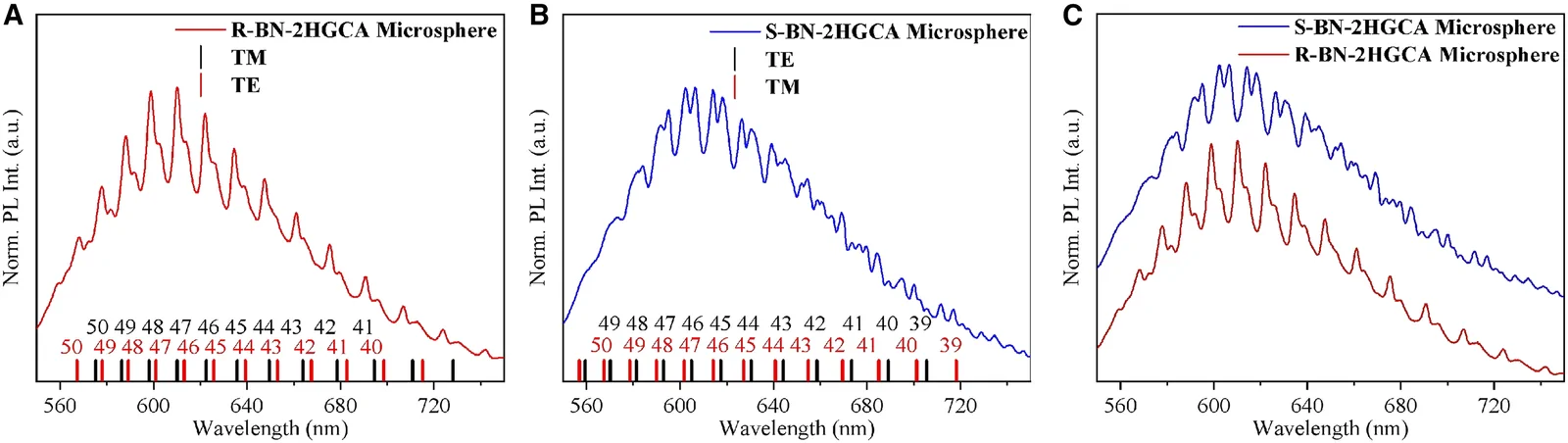
WGM mode analysis and performance (A and B) High-resolution μ-PL spectrum of a single microsphere with assignments for TE and TM modes. (C) μ-PL spectra of microspheres of (*R*)- and (*S*)-BN-2HGCA in same size.

A high-resolution μ-PL spectrum from a single microsphere is presented in Figures 5A and 5B, allowing for a detailed mode analysis. The WGM peaks can be classified into transverse electric (TE) and transverse magnetic (TM) polarization modes.^34^ These modes possess slightly different effective refractive indices and, thus, appear at distinct wavelengths. Based on theoretical calculations using Mie scattering theory^35^ and equations in S1 and S2, the primary mode numbers were assigned to the observed resonant peaks, as labeled in the figures. The performance of an optical resonator is defined by its Quality Factor (Q-factor), which represents the degree of light confinement and is inversely related to the optical loss. Figure 5C compares the μ-PL spectra of (*R*)- and (*S*)-enantiomer microspheres of a similar size. As expected for enantiomers, the WGM peak positions, spacing, and mode structure are nearly identical. The minor difference in the overall intensity is attributed to slight variations in excitation/collection alignment or particle positioning, not to an intrinsic molecular property. A Lorentzian fit on a representative peak (see the supplemental information for the simulation methods) yielded an average Q-factor of approximately 190. This modest Q-factor is consistent with several loss channels intrinsic to this emissive organic material, including residual self-absorption near the emission band, surface and boundary imperfections together with sphere polydispersity, and the disordered amorphous packing, all of which increase the round-trip optical loss; nonetheless, a Q of approximately 190 is sufficient to resolve the discrete WGM peaks and to establish the FSR–1/d scaling, and we do not claim a high-Q or lasing-grade cavity. We further note that the modest Q-factor is compatible with the suppression of the observable CPL; the suppression is a far-field averaging effect, in which emission from chiral emitters distributed throughout the sphere is scrambled by multiple internal reflections and geometric mode mixing before out-coupling, rather than a loss of the transverse-electric/transverse-magnetic polarization preserved by an individual mode over its round trips; stronger scattering and mode mixing, therefore, lower Q and enhance far-field depolarization simultaneously. This performance confirms that the self-assembled spheres completed the functional switch from molecular chirality to microscopic photonic resonance.^36^ This observation completes the functional switch: the “CPL-OFF” state, caused by the amorphous structure, simultaneously enabling the “WGM-ON” state.

**Figure 6:**
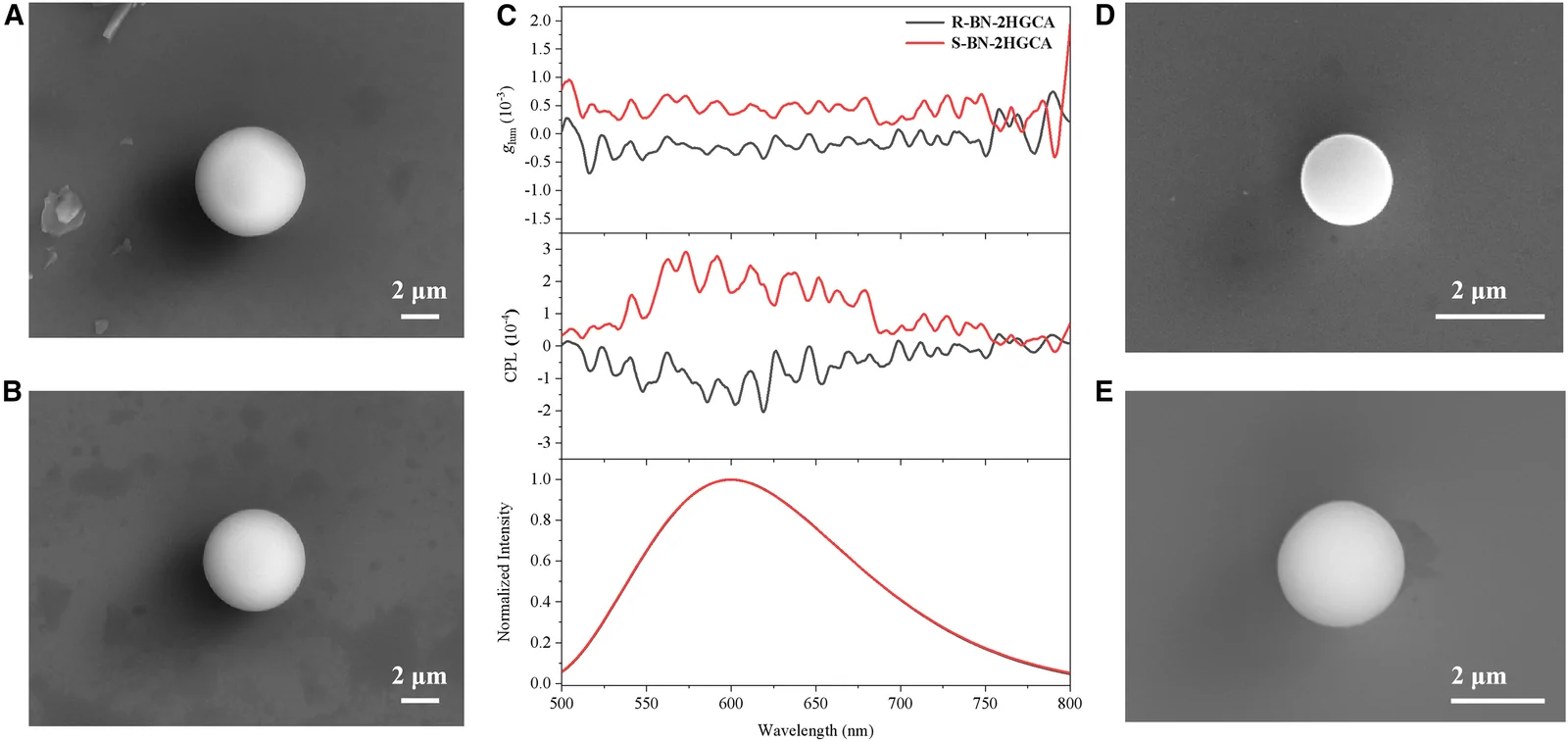
Stability of microspheres and CPL recovery in solution (A and B) SEM image of (A) (*R*)-enantiomer microspheres and (B) (*S*)-enantiomer microspheres. (C) CPL spectra of chloroform-dissolved (*R*)- and (*S*)-BN-2HGCA microspheres. (D and E) SEM image of (D) (*R*)-enantiomer microspheres and (E) (*S*)-enantiomer microspheres after dissolution and re-self-assembly.

After 12 months of storage (at ambient temperatures ranging from 5°C to 39°C), SEM characterization of the self-assembled microspheres (Figures 6A and 6B) demonstrated their preserved spherical morphology with excellent stability. The microspheres were dissolved in chloroform, and the resulting solution was analyzed by CPL spectroscopy, as shown in Figure 6C. The CPL signal was recovered, corresponding to a transition from microscopic photon resonance to molecular chirality. Using the dissolution solution for self-assembly, microspheres were obtained again as shown in Figures 6D and 6E.

The demonstration of WGM resonance subsequent to the suppression of CPL provides a clear illustration of an aggregation-state-dependent functional transition. The highly uniform and smooth boundaries of the self-assembled spheres are morphologically conducive to multiple internal reflections; however, the extent to which these reflections contribute to the observed depolarization has not been directly established. The primary cause of CPL suppression is attributed to the optically isotropic, disordered packing of the aggregate. This dual-modal switching between the “CPL-OFF” state and the “WGM-ON” state represents a platform for functional materials. This concept may, in the future, be explored for applications such as multi-level security and anti-counterfeiting.

## Discussion

In summary, this work reports a controllable functional switch in a novel axially chiral Schiff base, (*R*/*S*)-BN-2HGCA, where the dominant optical function is dictated by its physical aggregation state. In the dissolved molecular state, the compound is chiroptically active and exhibits clear CPL signals, with a *g*_lum_ value of approximately +4.5 × 10^−4^. Upon self-assembly into microspheres, this CPL function is completely suppressed, with the *g*_lum_ value dropping to the baseline noise level. Structural and optical investigations indicate that this suppression is associated with the optically isotropic, disordered packing of the aggregate, to which polarization randomization by multiple internal reflections within the spherical cavity may further contribute. It is demonstrated that this structural confinement, while causing the loss of chiroptical activity, simultaneously enables WGM resonance. The morphologically pristine spheres are demonstrated to be functional optical microcavities supporting clear WGM resonance. The WGM phenomenon was quantitatively confirmed by the linear relationship observed between the FSR and the inverse sphere diameter. Furthermore, an average Q-factor of approximately 190 was calculated from the high-resolution spectra. This study demonstrates a clear toggling from a “CPL-ON” state to a “WGM-ON” state, illustrating how physical aggregation and optical confinement can be directly harnessed to govern the dominant photonic function.

### Limitations of the study

The present study has several limitations. First, because the CPL signals are weak and close to the instrumental baseline, the absolute *g*_lum_ values should be interpreted cautiously; the conclusions, therefore, rely mainly on the reproducible sign reversal and mirror-image relationship of the two enantiomers. Second, the relative contributions of disordered molecular packing and cavity-induced polarization randomization to CPL suppression were not experimentally separated. Measurements in a refractive-index-matching medium would help distinguish these effects. Third, the observed WGM resonances exhibit a moderate average Q-factor of approximately 190, and lasing behavior was not investigated. Future studies should examine how particle size, surface quality, internal packing, and refractive-index contrast affect polarization preservation and cavity performance.

## Resource availability

### Lead contact

Requests for further information and resources should be directed to and will be fulfilled by the lead contact, Liang Tong (tongliang6363@jxust.edu.cn).

### Materials availability

All unique materials generated in this study, including (*R*)-BN-2HGCA and (*S*)-BN-2HGCA, and the corresponding self-assembled microspheres, are available from the lead contact upon reasonable request.

### Data and code availability

• All data reported in this paper will be shared by the lead contact upon request.
• This paper does not report original code.
• Any additional information required to reanalyze the data reported in this paper is available from the lead contact upon request.

## Acknowledgments

This study was supported by 10.13039/501100002858China Postdoctoral Science Foundation (2025M770125), 10.13039/501100004479Jiangxi Provincial Natural Science Foundation (20252BAC240243), Jiangxi Overseas Talent Program (20232BCJ25061), Jiangxi University of Science and Technology High-level Talent Research Project (205200100632 and 205200100543), 10.13039/501100019037Key Science and Technology Research Project in Jiangxi Province Department of Education (GJJ2200821), Jiangxi Provincial Key Laboratory of Functional Crystalline Materials Chemistry (2024SSY05161), the Jiangxi Provincial Key Laboratory of Functional Molecular Materials Chemistry (20212BCD42018), 10.13039/501100003382CREST (JPMJCR20T4), and 10.13039/501100020962ACT-X (JPMJAX23D8) from 10.13039/501100002241Japan Science and Technology Agency (JST), 10.13039/501100001691KAKENHI (JP24H00470, JP24H01693, JP20J00845, and JP24K17742) from 10.13039/501100001691Japan Society for the Promotion of Science (10.13039/501100001691JSPS), 10.13039/501100008662Murata Science and Education Foundation (M25AN123), and Toyo Gousei Foundation. The project was sponsored by Distinguished Professor Program of Jinggang Scholars in Institutions of Higher Learning.

## Author contributions

Conceptualization, L.T. and S.K.; methodology, Y.L.; investigation, Y.L.; data curation, J.L., Y.L., and C.C.; software, Y.L. and C.C.; validation, Y.L.; visualization, Y.L.; project administration, L.T. and S.K.; writing – original draft, Y.L. and L.T.; writing – review & editing, J.L., L.T., J.Q., S.K., and Y.Y.; funding acquisition, J.L., L.T., J.Q., S.K., and Y.Y.; resources, S.K. and Y.Y.; supervision, J.L., L.T., J.Q., S.K., and Y.Y.

## Declaration of interests

The authors declare no competing interests.

## Declaration of generative AI and AI-assisted technologies in the writing process

After writing the draft of our manuscript, we utilized large language models (LLMs, here Kimi and Grammarly) to correct grammar and scientific rigor. After using this tool, we reviewed and edited the content as needed and take full responsibility for the content of the published article.

## STAR★Methods

### Key resources table

**Table:** 

REAGENT or RESOURCE	SOURCE	IDENTIFIER
**Chemicals, peptides, and recombinant proteins**		

4-Methoxybenzaldehyde	Titan Technology Exploration Platform	CAS: 123-11-5
4-Hydroxyphenylacetonitrile	Titan Technology Exploration Platform	CAS: 14191-95-8
Sodium hydroxide	Titan Technology Exploration Platform	CAS: 1310-73-2
Ethanol	Titan Technology Exploration Platform	CAS: 64-17-5
Hydrochloric acid	Titan Technology Exploration Platform	CAS: 7647-01-0
Sodium bicarbonate	Titan Technology Exploration Platform	CAS: 144-55-8
Petroleum ether	Titan Technology Exploration Platform	CAS: 8032-32-4
Dichloromethane	Titan Technology Exploration Platform	CAS: 75-09-2
Hexamethylenetetramine	Titan Technology Exploration Platform	CAS: 100-97-0
Acetic acid	Titan Technology Exploration Platform	CAS: 64-19-7
(R)-(+)-1,1′-Binaphthyl-2,2′-diamine	Titan Technology Exploration Platform	CAS: 18741-85-0
(S)-(−)-1,1′-Binaphthyl-2,2′-diamine	Titan Technology Exploration Platform	CAS: 18531-95-8
Methanol	Titan Technology Exploration Platform	CAS: 67-56-1
Chloroform	Titan Technology Exploration Platform	CAS: 67-66-3
Sodium dodecyl sulfate (SDS)	Titan Technology Exploration Platform	CAS: 151-21-3
N,N-Dimethylformamide (DMF)	Titan Technology Exploration Platform	CAS: 68-12-2
Tetrahydrofuran (THF)	Titan Technology Exploration Platform	CAS: 109-99-9
Toluene	Titan Technology Exploration Platform	CAS: 108-88-3
Acetone	Titan Technology Exploration Platform	CAS: 67-64-1
1,4-Dioxane	Titan Technology Exploration Platform	CAS: 123-91-1
Potassium bromide (KBr)	Titan Technology Exploration Platform	CAS: 7758-02-3
Deuterochloroform (CDCl3)	Titan Technology Exploration Platform	CAS: 865-49-6
HCA	This paper	N/A
HGCA	This paper	N/A
(R)-BN-2HGCA	This paper	N/A
(S)-BN-2HGCA	This paper	N/A
Self-assembled (R)-BN-2HGCA microspheres	This paper	N/A
Self-assembled (S)-BN-2HGCA microspheres	This paper	N/A

**Software and algorithms**		

Microsoft Excel (WGM simulation, Lorentzian fitting, linear regression, and Q-factor calculation)	Microsoft	RRID: SCR_016137

**Other**		

Scanning electron microscope	Hitachi	Model: SU-8000
Scanning electron microscope	Philips	Model: XL30W/TMP
Upright optical microscope	Olympus	Model: BX53
UV–visible spectrophotometer	Shimadzu	Model: U-2550
Fluorescence spectrophotometer	Hitachi	Model: F-4600
Spectropolarimeter	JASCO	Model: J-1500
CPL spectrophotometer	JASCO	Model: CPL-300
NMR spectrometer	Bruker	Model: Avance 400

### Experimental model and study participant details

This study did not involve animals, human participants, plants, microbial strains, cell lines, primary cell cultures, or other biological experimental models or study participants. Therefore, the reporting requirements related to species or strain, genotype, developmental stage, sex or gender, maintenance or care, institutional permissions and oversight, cell-line authentication, mycoplasma testing, human sample size, and group allocation are not applicable. Accordingly, no assessment of the influence of sex or gender was applicable to this materials-science study.

### Method details

#### Materials and instrumentation

All chemicals and reagents were purchased from the Titan Technology Exploration Platform and used as received. The morphology of the microspheres was examined by scanning electron microscopy (SEM; Hitachi SU-8000 and Philips XL30W/TMP) at an operating voltage of 15 kV. Optical and fluorescence microscopy images were acquired using an Olympus BX53 upright microscope. UV–visible absorption spectra were recorded using a Shimadzu U-2550 spectrophotometer with 1 cm and 0.1 mm quartz cuvettes for solution and aggregate samples, respectively. Fluorescence spectra were recorded using a Hitachi F-4600 fluorescence spectrophotometer at 300 V with excitation and emission slit widths of 10 nm and a scan rate of 2400 nm min^−1^. CD spectra were recorded using a JASCO J-1500 spectropolarimeter under thermostated conditions. Samples in 10 mm quartz cells were purged with N_2_ for 30 min before scanning over 200–800 nm at 200 nm min^−1^. Each CD spectrum represents the average of three consecutive scans. CPL spectra were recorded using a JASCO CPL-300 spectrophotometer in 1 mm quartz cuvettes over 400–800 nm at 200 nm min^−1^, with an excitation wavelength of 365 nm and a DC voltage of approximately 0.5 V. CPL measurements were repeated three to five times. ^1^H NMR spectra were recorded using a Bruker Avance 400 spectrometer with samples dissolved in CDCl_3_.

#### Synthesis of HCA

4-Methoxybenzaldehyde and 4-hydroxyphenylacetonitrile were added to a 250 mL flask and reacted at 50°C for 24 h in a sodium hydroxide solution in anhydrous ethanol. After cooling to room temperature, ethanol was removed by vacuum distillation. The mixture was acidified with excess 10% hydrochloric acid and subsequently neutralized with excess sodium bicarbonate. After dilution with purified water, the organic phase was extracted and concentrated under reduced pressure. The crude product was purified by column chromatography using petroleum ether/dichloromethane (3:1, v/v) as the eluent to afford HCA.

#### Synthesis of HGCA

HCA and hexamethylenetetramine were added to a 100 mL flask at a molar ratio of 1:4, followed by 30 mL of acetic acid. The reaction was monitored by thin-layer chromatography until completion. After cooling to room temperature, sodium bicarbonate and a small amount of water were slowly added until gas evolution ceased. The product was extracted with dichloromethane, and the organic layer was concentrated under reduced pressure. The crude product was purified by column chromatography using dichloromethane as the eluent to afford HGCA.

#### Synthesis of (R/S)-BN-2HGCA

HGCA and (R/S)-[1,1′-binaphthalene]-2,2′-diamine were added to a 100 mL flask at a molar ratio of 2:1. Methanol and two drops of glacial acetic acid were added, and the mixture was reacted at 65°C for 12 h. Reaction progress was monitored by thin-layer chromatography. After completion, the solvent was removed under reduced pressure. The residue was dissolved in dichloromethane, followed by the dropwise addition of petroleum ether to induce recrystallization and afford (R/S)-BN-2HGCA.

#### Preparation of microspheres

Microspheres were prepared using a miniemulsion method. (R/S)-BN-2HGCA was dissolved in chloroform at 10 mg mL^−1^. Two milliliters of an SDS solution at a concentration of 10 mmol L^−1^ were placed in a 5 mL vial. Subsequently, 0.1 mL of the (R/S)-BN-2HGCA solution was added dropwise, and the mixture was homogenized at 10,000 rpm until complete emulsification. After standing for 72 h at 25°C, the precipitated microspheres were collected and washed with distilled water.

#### WGM simulation and spectral analysis

The WGM emission was simulated using Equations S1 and S2 for transverse electric (TE) and transverse magnetic (TM) mode emissions, respectively. An underlying broad emission based on the μ-PL measurements was assumed and superimposed with the calculated WGMs. The mode positions were calculated using Equations S1 and S2, while the individual peak shapes were approximated using Lorentzian functions of appropriate height and width and weighted by the underlying PL intensity. Within the uncertainty of the microsphere radius determined by optical microscopy, the radius was adjusted so that the calculated TE and TM modes agreed with the experimental mode positions. Because the refractive index of the π-conjugated material depends on wavelength, the average refractive-index values obtained by spectroscopic ellipsometry were used for the (R)- and (S)-BN-2HGCA microspheres. Microsoft Excel was used for the WGM simulations, Lorentzian fitting, linear regression, and Q-factor calculations.

### Quantification and statistical analysis

No inferential statistical tests were performed. CD spectra represent the average of three consecutive scans, and CPL measurements were repeated three to five times. The reported glum values were averaged over the 550-700 nm emission range. Microsphere size distributions are presented as mean ± standard deviation. Free spectral range values were obtained from the spacings between adjacent WGM peaks and plotted against the inverse microsphere diameter. Linear regression was performed using Microsoft Excel. Q factors were calculated as Q = λ/Δλ, where Δλ was obtained from Lorentzian fitting of representative WGM peaks in Microsoft Excel. Unless otherwise stated, no data points were excluded from the analyses.

## Supplemental information

Document S1. Figures S1–S10, Table S1, and Schemes S1 and S2
