# Supplementary material for: Functional switching from circularly polarized luminescence to whispering gallery modes in a chiral Schiff base
Source: iScience. 2026 Aug 29;29(9):117379. doi: 10.1016/j.isci.2026.117379 (PMC13551943; doi:10.1016/j.isci.2026.117379)
Supplement: Supplementary file 1 [file mmc1.pdf]

iScience, Volume *29*

Supplemental information

Functional switching from circularly polarized luminescence to whispering gallery modes in a chiral Schiff base

Jinhui Li, Yue Luo, Cong Chen, Liang Tong, Jiabin Qiu, Soh Kushida, and Yohei Yamamoto

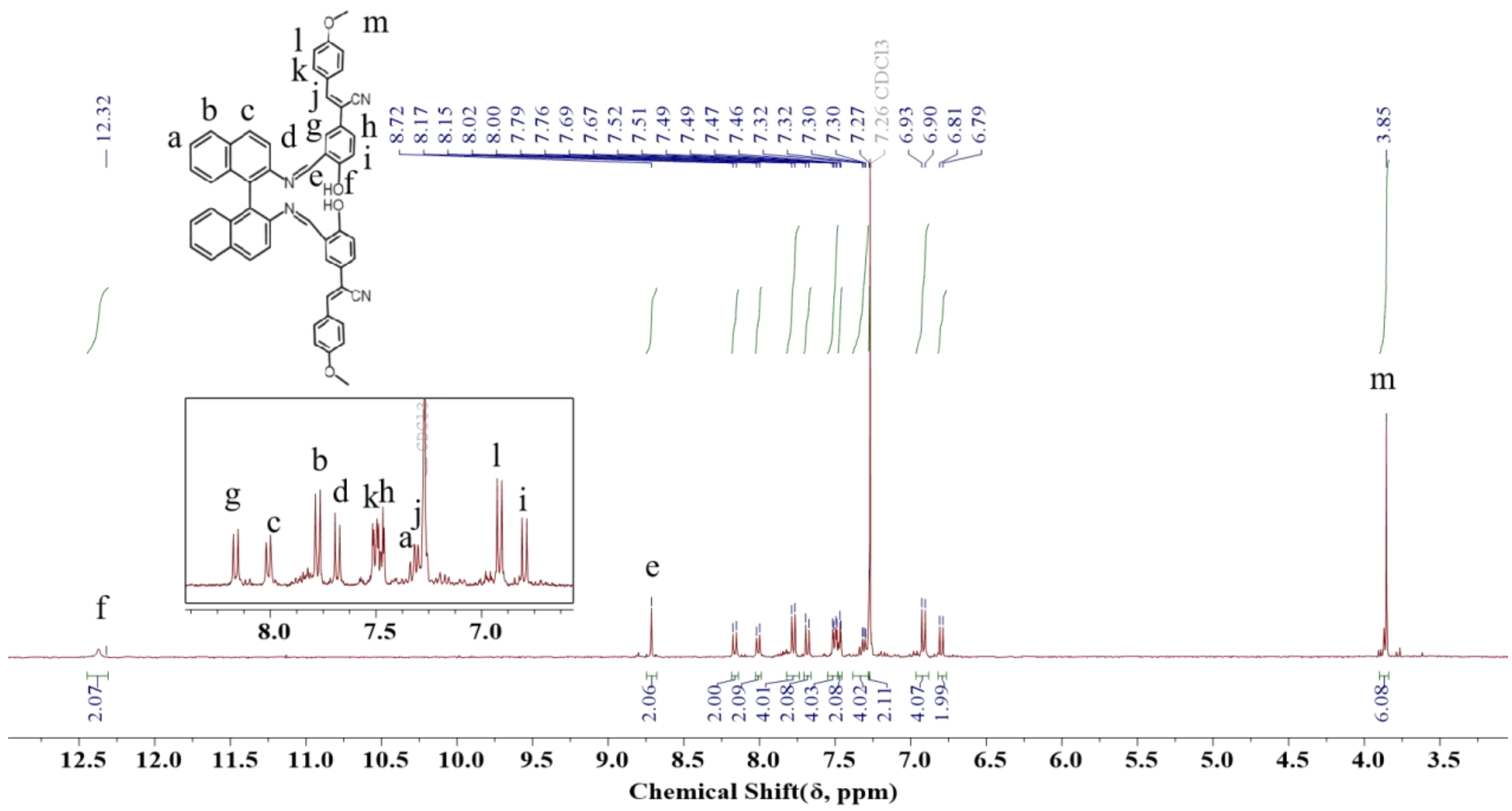

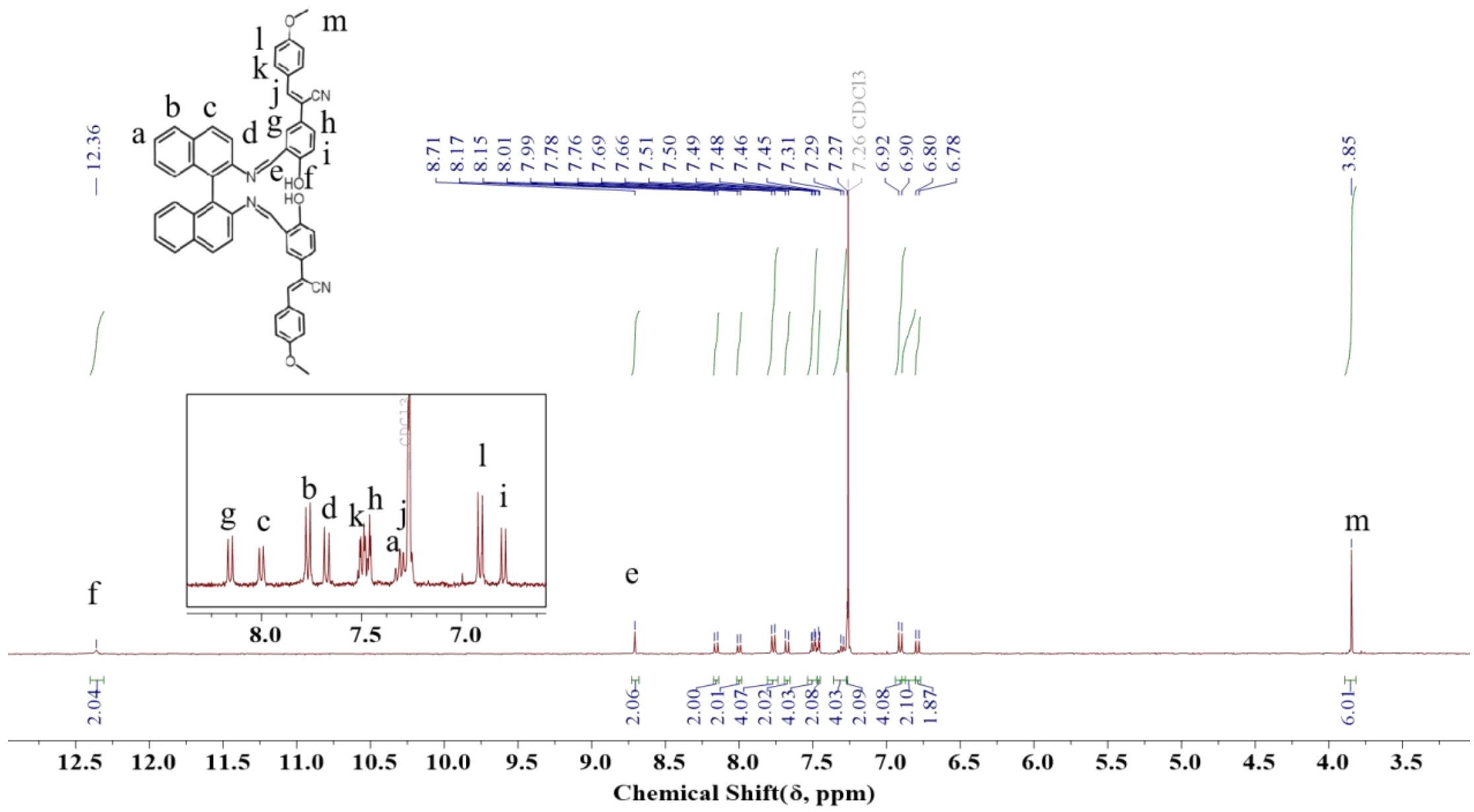

Figure S1. ¹H NMR spectra of (R/S)-BN-2HGCA.

R-BN-2HGCA: According to its ¹H NMR spectrum, the signals in the chemical-shift range δ 8.00–7.27 ppm correspond to the characteristic protons of the naphthalene rings. The doublets observed at δ 7.46 and 6.79 ppm are assigned to the protons on the benzene rings. The singlets at δ 12.32 and 8.72 ppm are attributable to the phenolic -OH and the N=CH protons, respectively. Detailed data: ¹H NMR (400 MHz, $CDCl_3$) δ 12.32 (s, 2H, –OH), 8.72 (s, 2H, N=CH), 8.15 (d, J = 8.8 Hz, 2H,

ArH), 8.00 (d, J = 8.3 Hz, 2H, ArH), 7.76 (d, J = 8.9 Hz, 4H, ArH), 7.69 (d, J = 8.9 Hz, 2H, ArH), 7.51 (dd, J = 8.6, 2.6 Hz, 4H, ArH), 7.46 (d, J = 2.4 Hz, 2H, ArH), 7.32–7.30 (m, 4H, ArH), 7.27 (s, 2H, –C=CH), 6.90 (d, J = 8.9 Hz, 4H, ArH), 6.79 (d, J = 8.7 Hz, 2H, ArH), 3.85 (s, 6H, –CH).

S-BN-2HGCA: Its ^{1}H NMR spectrum shows the same characteristic regions: signals between δ 8.00–7.27 ppm for the naphthalene protons, doublets at δ 7.46 and 6.78 ppm for the benzene-ring protons, and singlets at δ 12.36 and 8.71 ppm for the phenolic –OH and N=CH protons, respectively. Detailed data: ^{1}H NMR (400 MHz, $CDCl_3$) δ 12.36 (s, 2H, –OH), 8.71 (s, 2H, N=CH), 8.15 (d, J = 8.7 Hz, 2H, ArH), 8.01 (d, J = 8.2 Hz, 2H, ArH), 7.78 (d, J = 8.8 Hz, 4H, ArH), 7.69 (d, J = 8.8 Hz, 2H, ArH), 7.49 (dd, J = 8.6, 2.3 Hz, 4H, ArH), 7.46 (d, J = 2.4 Hz, 2H, ArH), 7.31–7.29 (m, 4H, ArH), 7.27 (s, 2H, –C=CH), 6.92 (d, J = 8.9 Hz, 4H, ArH), 6.78 (d, J = 8.7 Hz, 2H, ArH), 3.85 (s, 6H, –CH).

In summary, every proton in the (R/S)-BN-2HGCA structure is accounted for in the respective spectra, and the integrated peak areas are consistent with the expected proton counts.

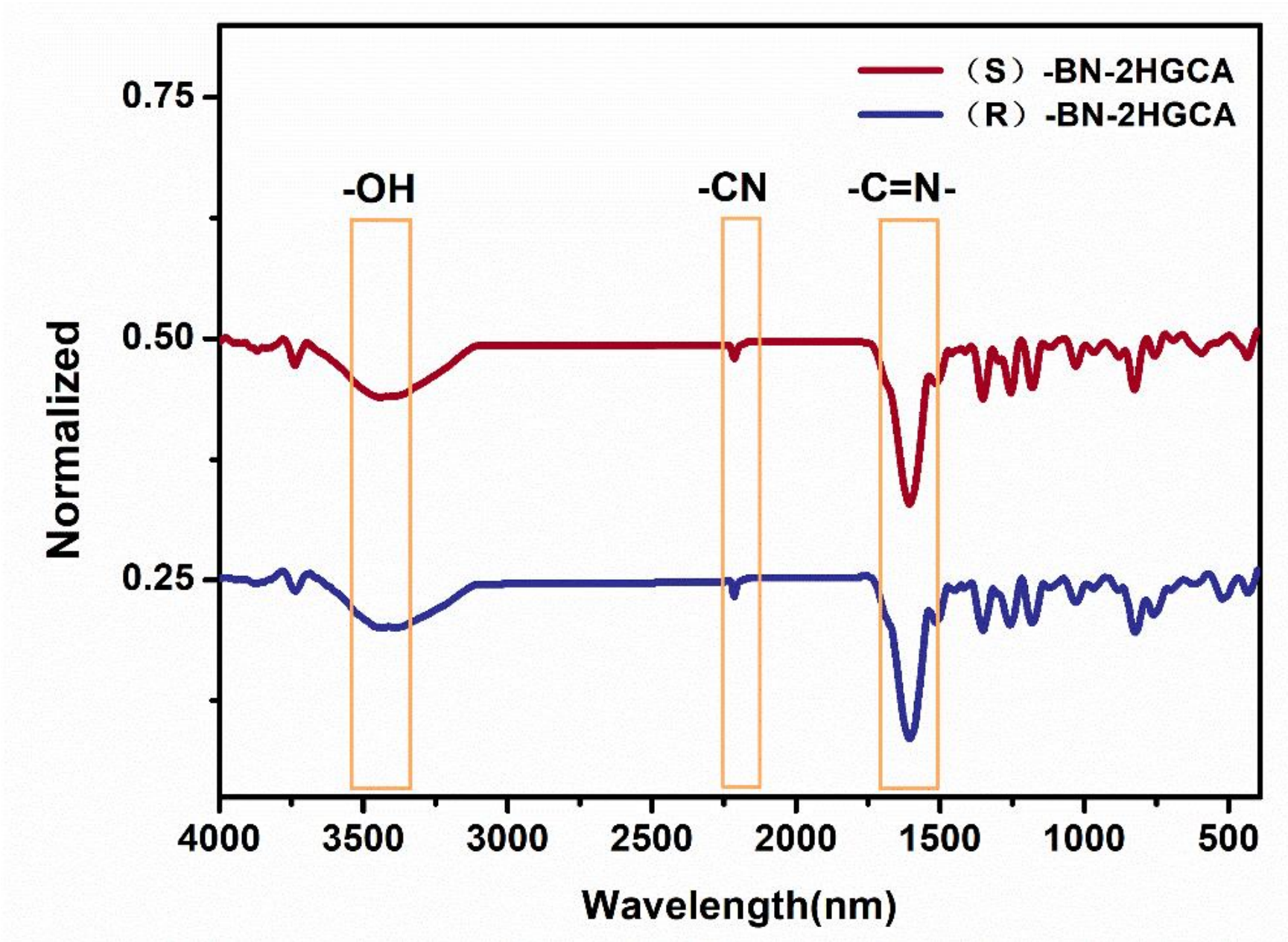

Figure S2. FT-IR spectra of (R/S)-BN-2HGCA.

To further confirm the functional groups present in the target product, (R/S)-BN-2HGCA were characterized by FT-IR spectroscopy; the spectra are shown in the figure. After condensation, the aldehyde C-H stretching vibrations characteristic of HGCA (at 2820 and 2720 cm^{-1}) have completely disappeared, while a new C=N stretching band assigned to the imine in (R/S)-BN-2HGCA appears at 1600 cm^{-1}. In addition, a nitrile (C≡N) stretching vibration is observed at 2217 cm^{-1}, and the O-H stretching band of the phenolic hydroxyl groups is visible between 3500 and 3200 cm^{-1}. These data demonstrate that the desired product has been successfully obtained via Schiff-base condensation.

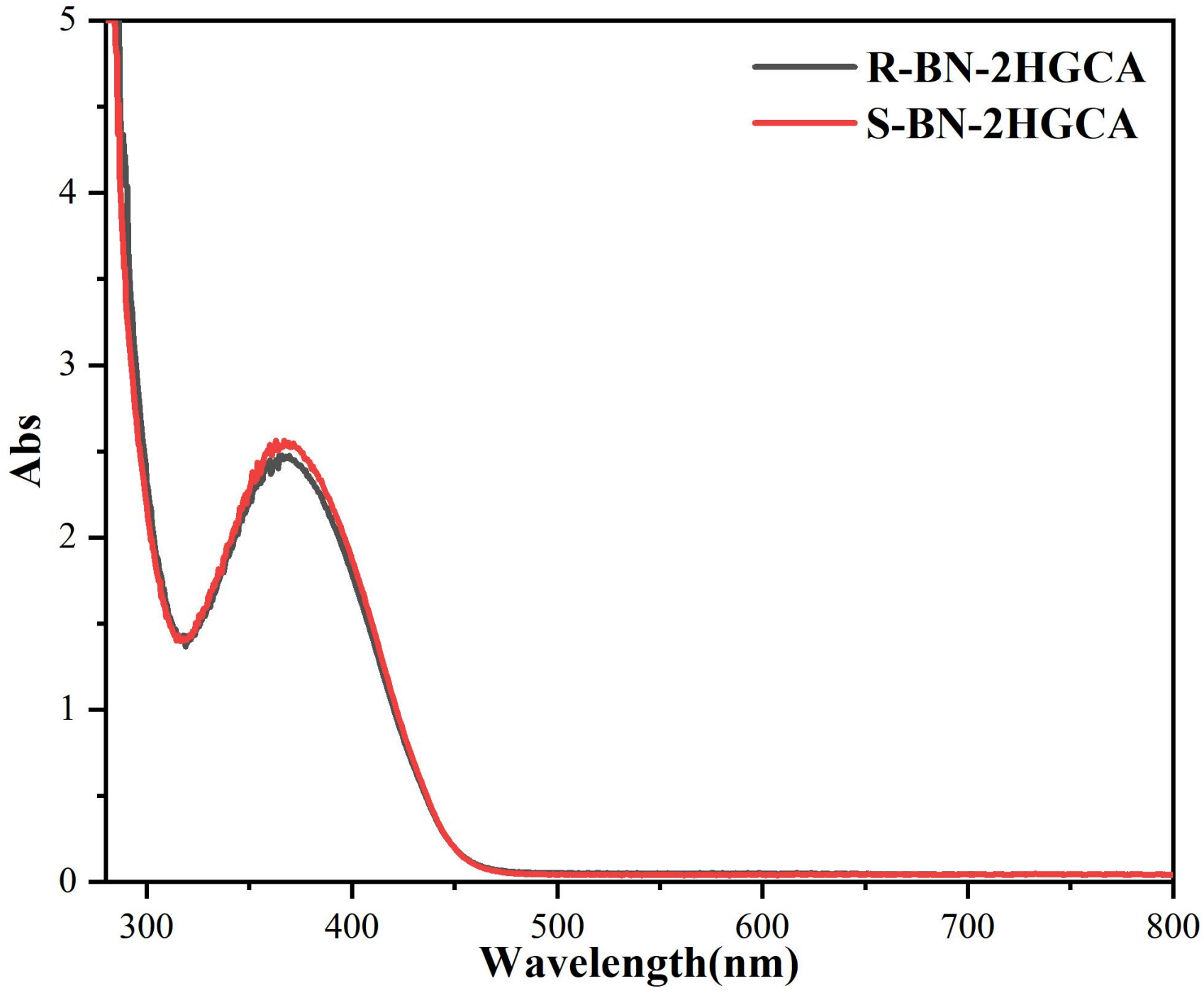

Figure S3. UV–visible absorption spectra of (R/S)-BN-2HGCA in DMF.

In pure DMF solvent, (R/S)-BN-2HGCA exhibit maximum absorption peaks at a wavelength of 367 nm.

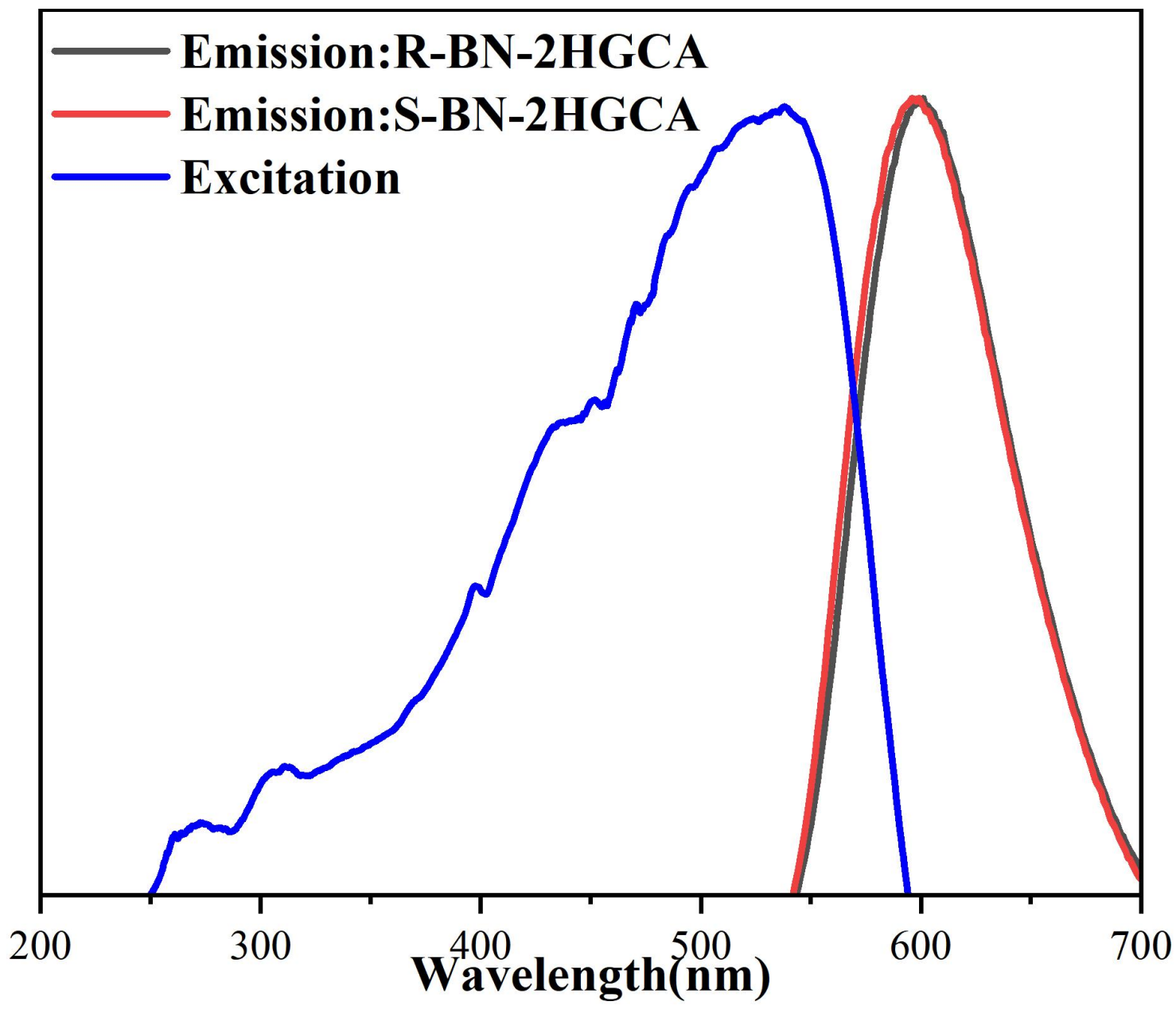

Figure S4. Excitation and emission spectra of (R/S)-BN-2HGCA in chloroform.

Upon dissolving (R/S)-BN-2HGCA in chloroform and conducting fluorescence testing, it was found to exhibit broadened absorption characteristics alongside a single-peak fluorescence emission profile, emitting at 620 nm.

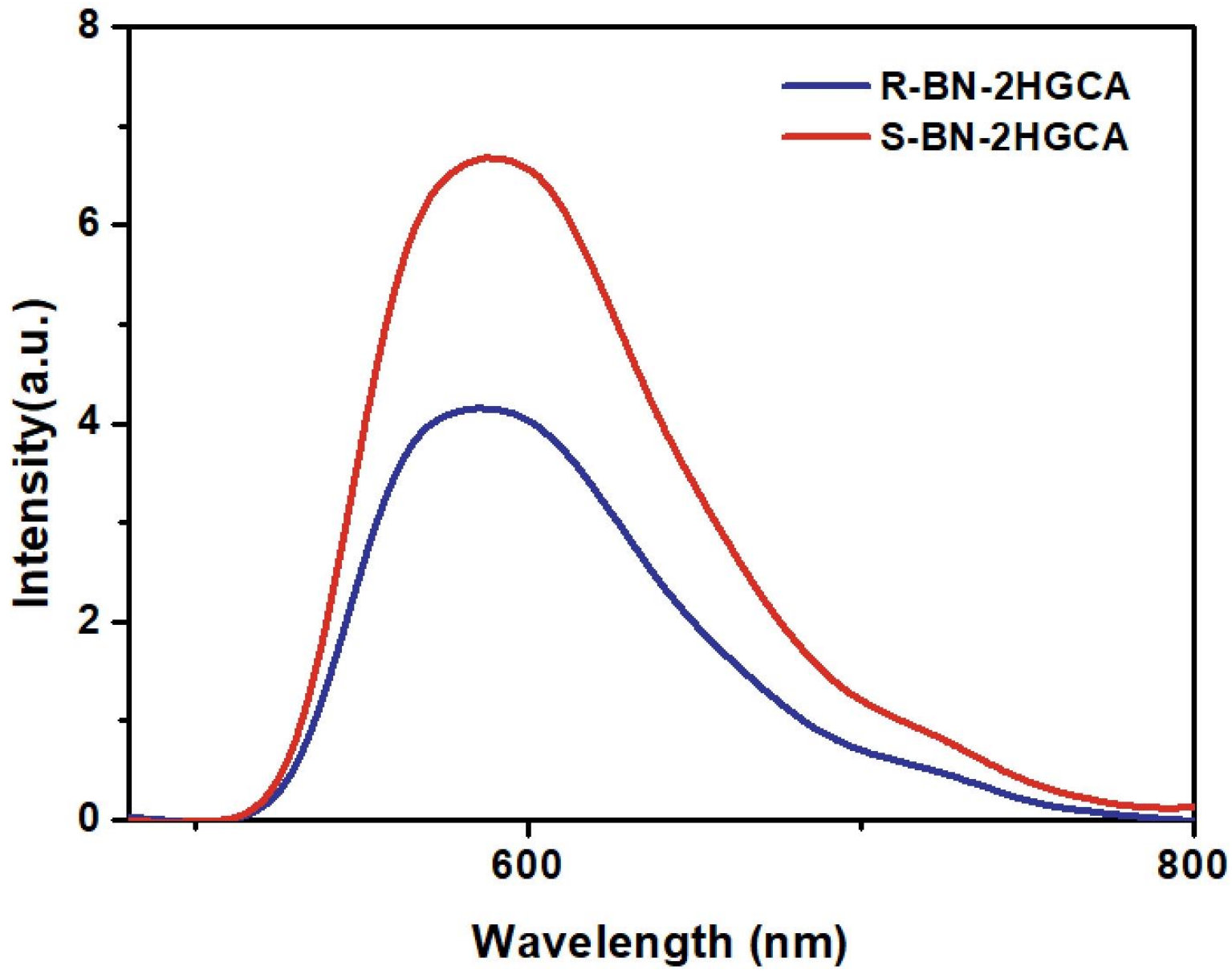

Figure S5. PL spectra of (R/S)-BN-2HGCA in THF (1 × 10−5 mol L−1).

Dissolve (R/S)-BN-2HGCA in THF to prepare 1×10^{-5} mol/L solutions for fluorescence testing. The fluorescence intensity peak of S-BN-2HGCA is higher than that of R-BN-2HGCA, indicating that the S-configured compound exhibits stronger fluorescence emission capability.

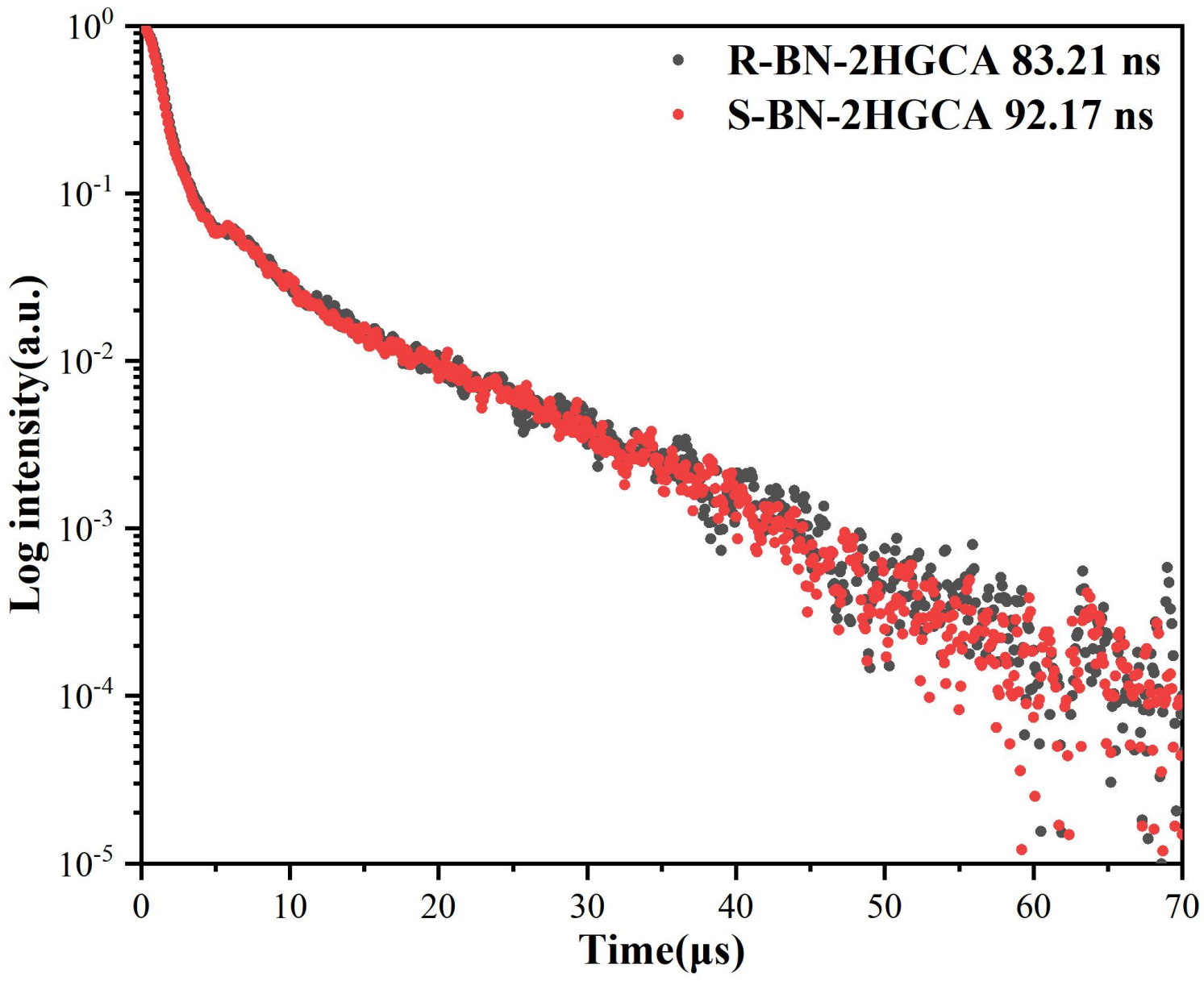

Figure S6. Fluorescence-lifetime decay curves (λex = 405 nm) of (R/S)-BN-2HGCA.

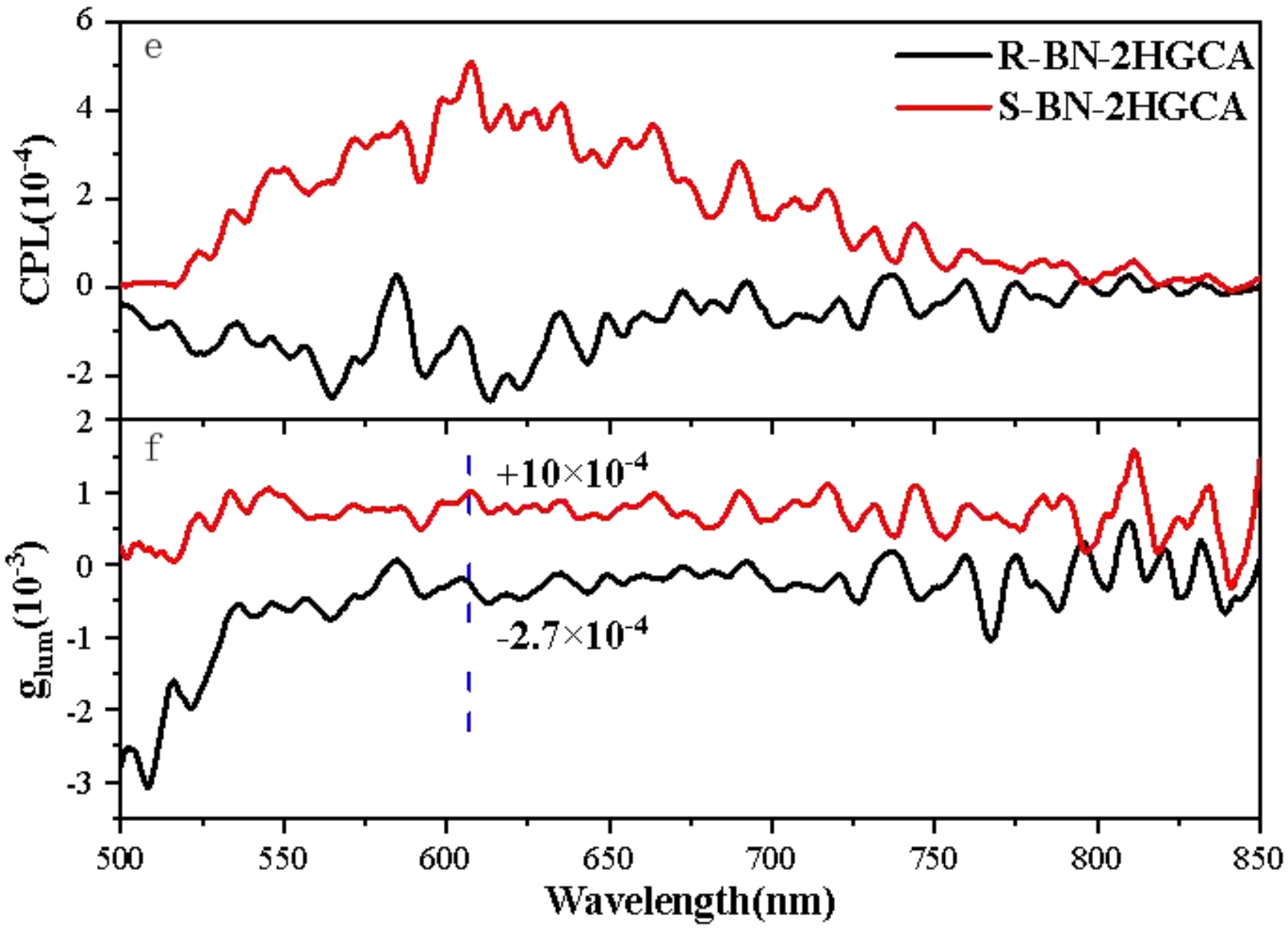

Figure S7. CPL spectra and corresponding glum spectra of (R/S)-BN-2HGCA in KBr solid.

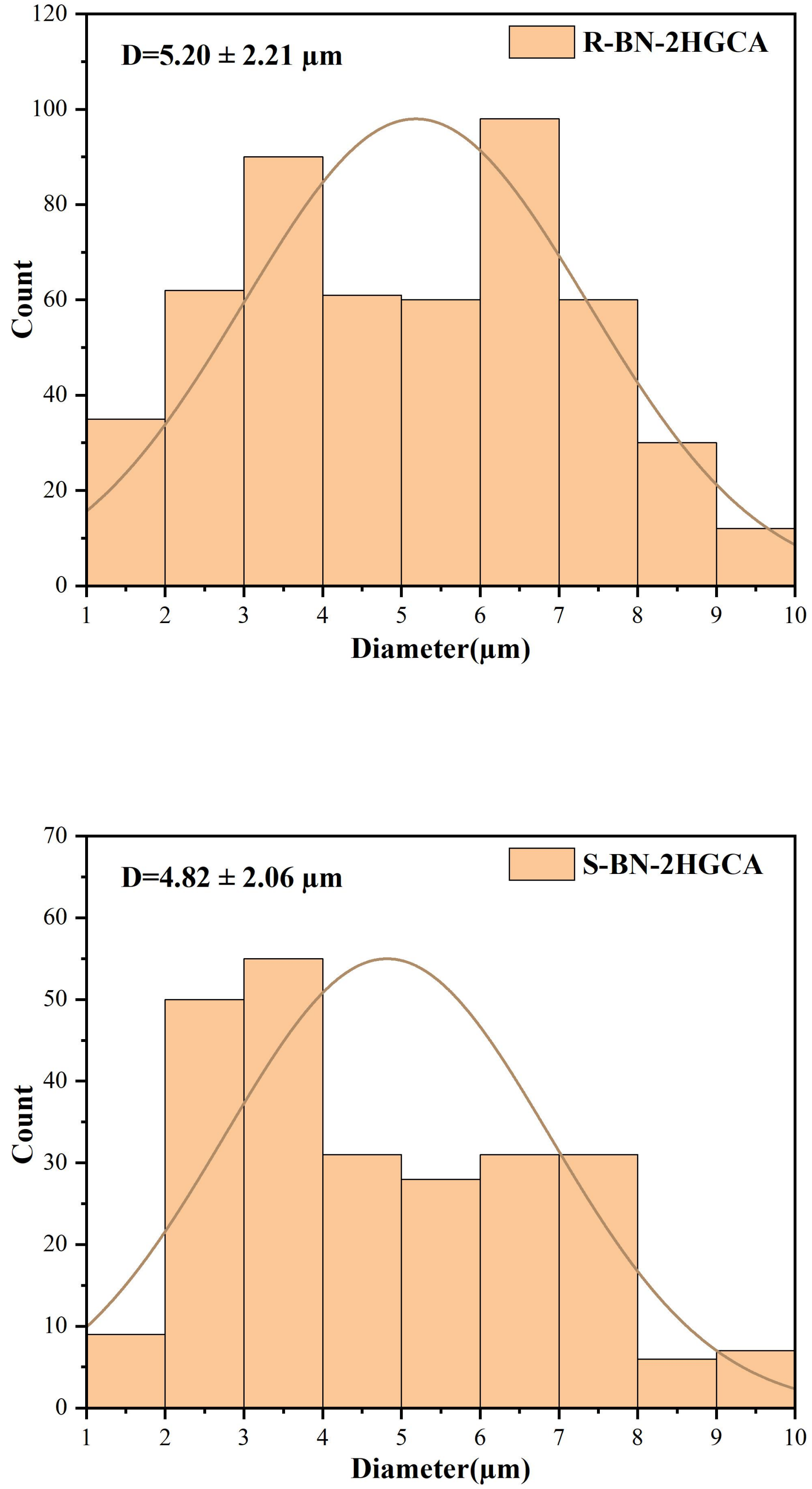

Figure S8. Particle-size distributions of self-assembled (R/S)-BN-2HGCA microspheres.

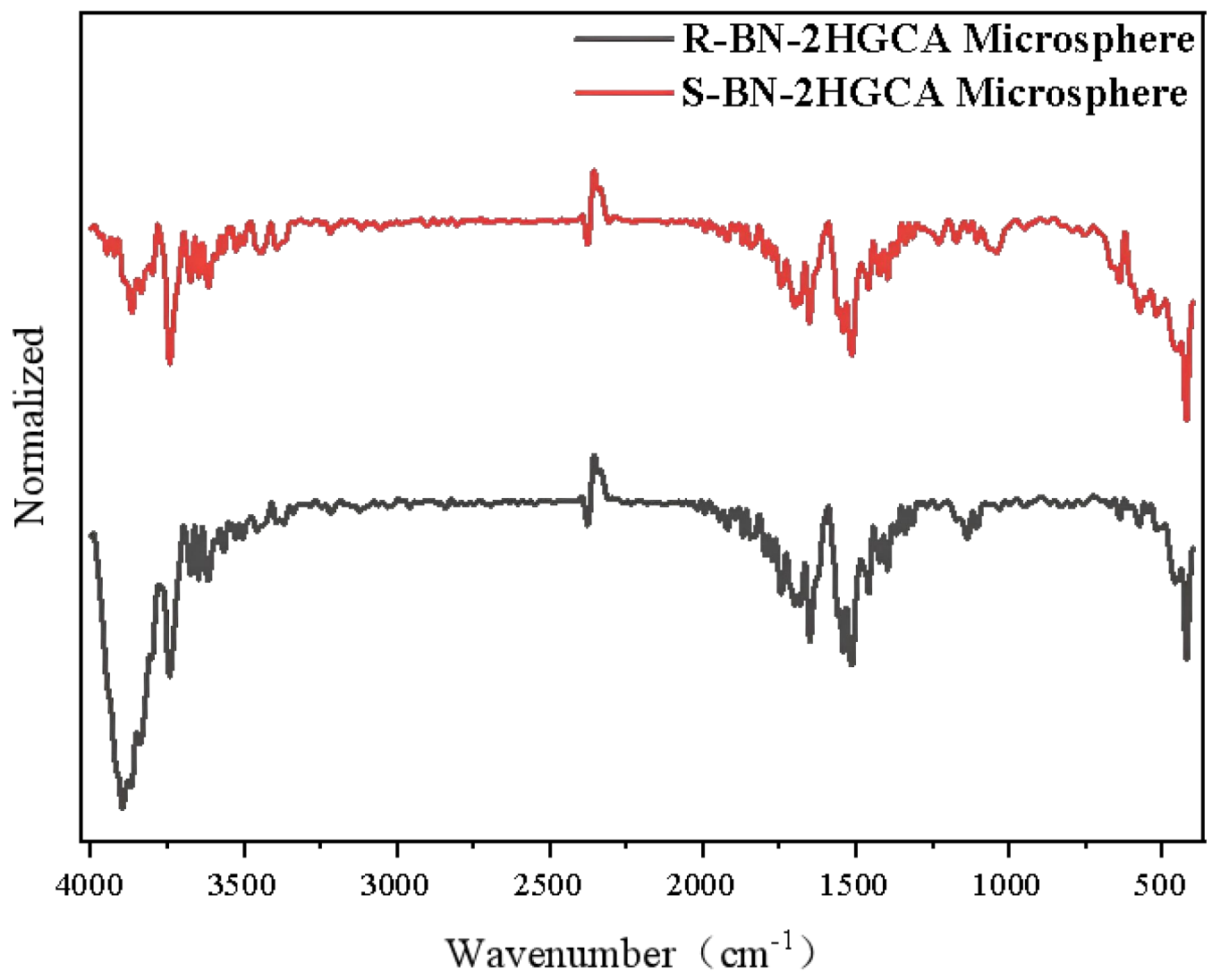

Figure S9. FT-IR spectra of (R/S)-BN-2HGCA microspheres.

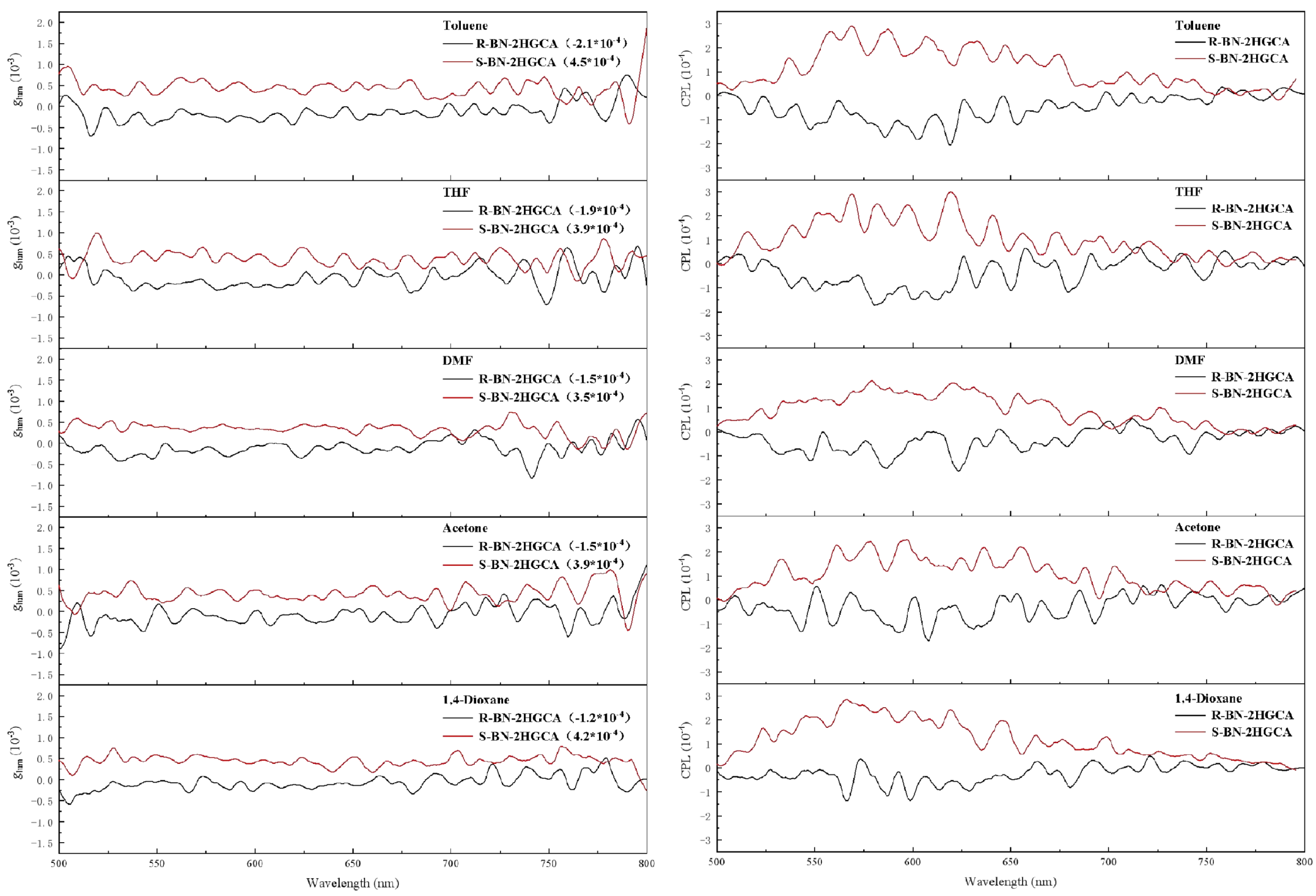

Figure S10. CPL spectra of (R/S)-BN-2HGCA in different solvents.

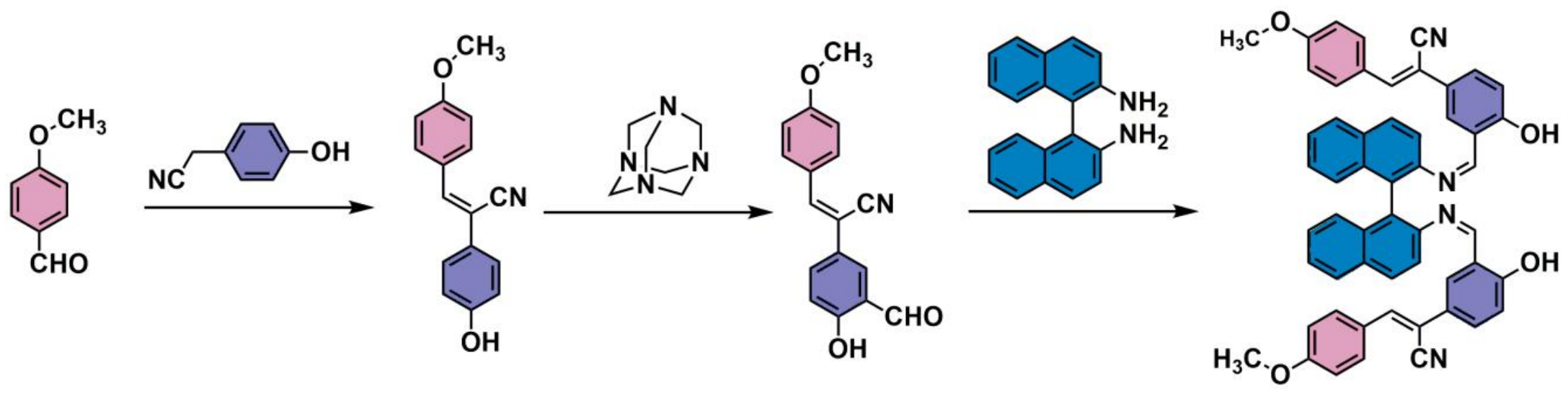

Scheme S1. Synthetic route to (R/S)-BN-2HGCA.

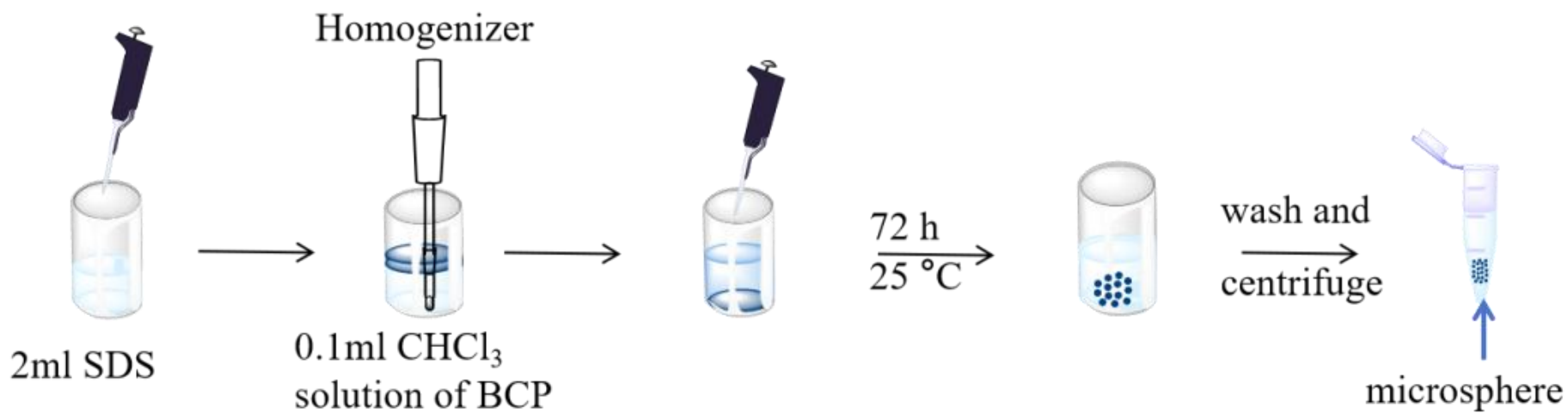

Scheme S2. Preparation of (R/S)-BN-2HGCA microspheres.

Table S1. Comparison of glum values for representative binaphthyl/BINOL-based CPL emitters.

Compound (system)	State	$\|g_{lum}\|$
(R/S)-BN-2HGCA (this work)	Methanol solution	4.5×10^{-4}
BINOL-BODIPY dyad (BDP1)[1]	DCM solution	$4.1–6.3 \times 10^{-5}$
R-BINOL-cyanostilbene (R-BINC-Al)[2]	THF solution	4.3×10^{-4}
R-BINOL-cyanostilbene (R-BINC-Ch)[2]	Mesophase	1.53×10^{-2}
Camphanic-biscyanostilbene[3]	Mesophase / Suspension	1.9×10^{-2} / 1.8×10^{-2}

Simulations

The simulations of the WGM emission are conducted using equations S1 and S2 for transverse electric (TE) and magnetic (TM) mode emissions, respectively[4].

$$\omega_n^E=\frac{c}{\alpha(\epsilon\mu)^{1/2}}\left[n+\frac{1}{2}+1.85576\left(n+\frac{1}{2}\right)^{\frac{1}{3}}-\frac{1}{\epsilon}\left(\frac{\epsilon\mu}{\epsilon\mu-1}\right)^{\frac{1}{2}}+\frac{3}{10\sqrt[3]{4}}1.8557^2\left(n+\frac{1}{2}\right)^{-\frac{1}{3}}-1.8557\left(n+\frac{1}{2}\right)^{-\frac{2}{3}}\frac{\epsilon^{\frac{3}{2}}}{3\sqrt[3]{2}(\epsilon-1)^{\frac{3}{2}}}\right] \quad \text{S1}$$

$$\omega_n^H=\frac{c}{\alpha(\epsilon\mu)^{1/2}}\left[n+\frac{1}{2}+1.85576\left(n+\frac{1}{2}\right)^{\frac{1}{3}}-\frac{1}{\mu}\left(\frac{\epsilon\mu}{\epsilon\mu-1}\right)^{\frac{1}{2}}+\frac{3}{10\sqrt[3]{4}}1.8557^2\left(n+\frac{1}{2}\right)^{-\frac{1}{3}}-1.8557\left(n+\frac{1}{2}\right)^{-\frac{2}{3}}\frac{3\epsilon-2}{3\sqrt[3]{2}\sqrt{\epsilon}(\epsilon-1)^{\frac{3}{2}}}\right] \quad \text{S2}$$

with $\lambda_n^{E,H} = 2\pi c/ \omega_n^{E,H}$, where ω_n^E (λ_n^E) and ω_n^H (λ_n^H) are frequencies (wavelengths) of the n-th TE and TM mode photoemission, respectively, ε (= η^2) is dielectric permittivity, μ (= 1) is the magnetic permeability, c is the speed of light, and a is the sphere's radius. For a simulation of the spectrum, an underlying broad emission, according to μ-PL measurements, was assumed with superimposed WGMs. The peak width in the simulated curves was chosen to roughly reproduce the experimental lines. Those modes were calculated using the equations S1 and S2, while their shape was approximated with a Lorentzian function of appropriate height and width, weighed with the intensity of the undelying PL. Within the margin of error of the sphere's radius (given by optical microscopy), the simulation adapts the radius so that both TE and TM modes agree well for given orders n, effectively determining the radius with much higher precision. Because η of π-conjugated depends on the wavelength, the average η values obtained by spectroscopic ellipsometry measurements were used for R- and S-BN-2HGCA .

Supplemental references

1. 1. Z. Liu, Z. Jiang, C. He, Y. Chen, Z. Guo, Dyes Pigm. 181 (2020) 108593, https://doi.org/10.1016/j.dyepig.2020.108593.
2. 2. H. Feng, M. Li, S. Wang, Y.-S. Feng, J. Mol. Struct. 1320 (2025) 139726, https://doi.org/10.1016/j.molstruc.2024.139726.
3. 3. S. Chen, L. Lin, S. Zheng, H. Guo, F. Yang, J. Mol. Liq. 391 (2023) 123176, https://doi.org/10.1016/j.molliq.2023.123176.
4. 4. N. Tanji, H. Yamagishi, K. Fujita, Y. Yamamoto, ACS Appl. Polym. Mater. 4 (2022) 1065–1070, https://doi.org/10.1021/acsapm.1c01500.
